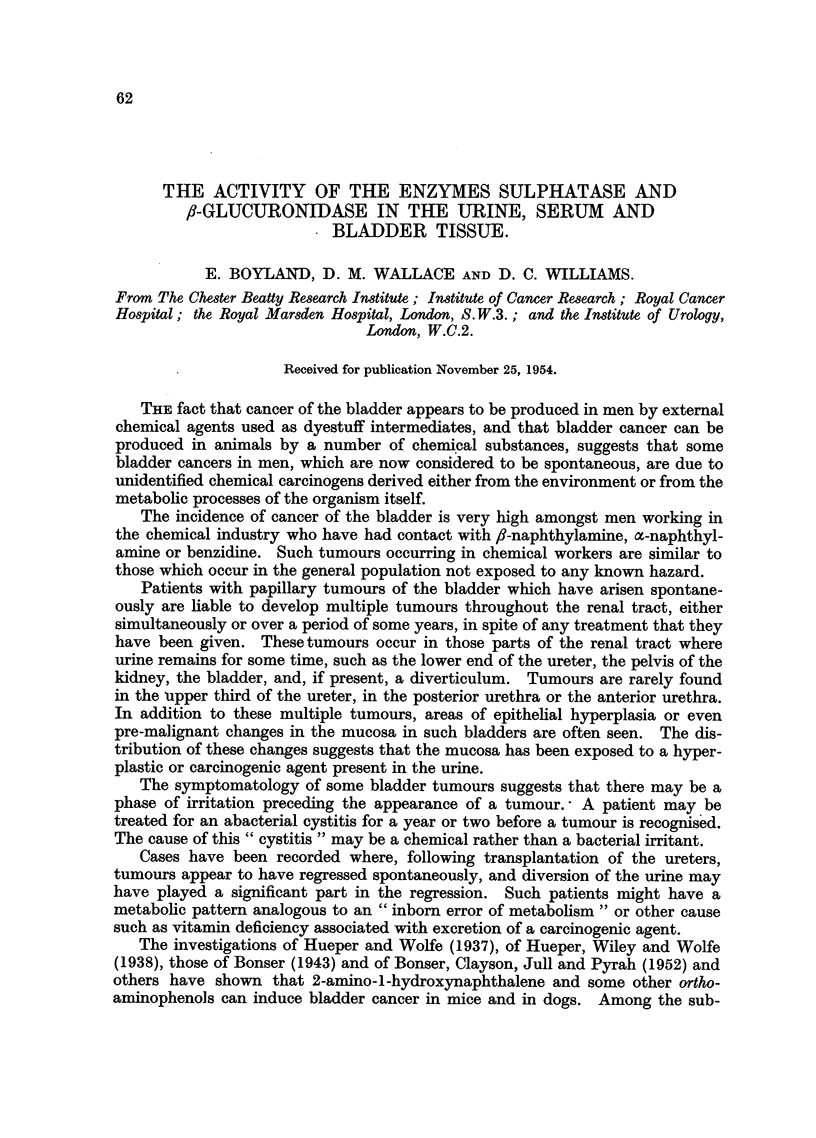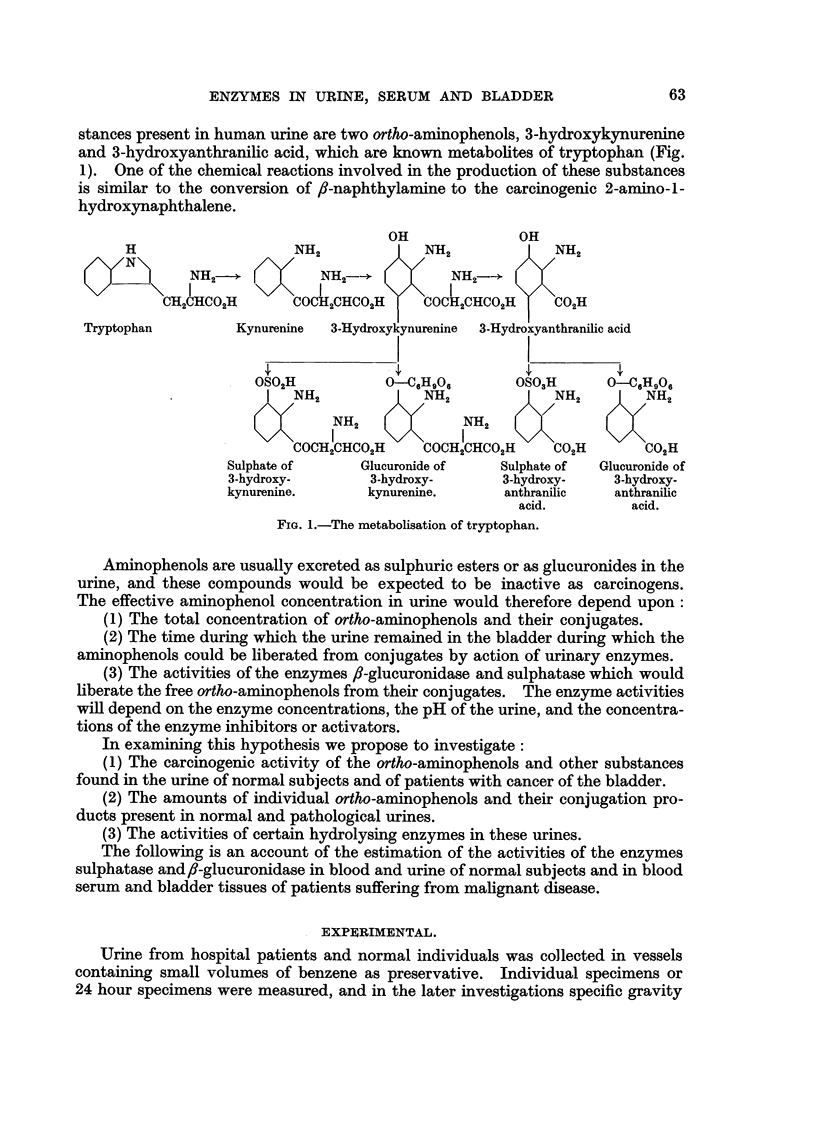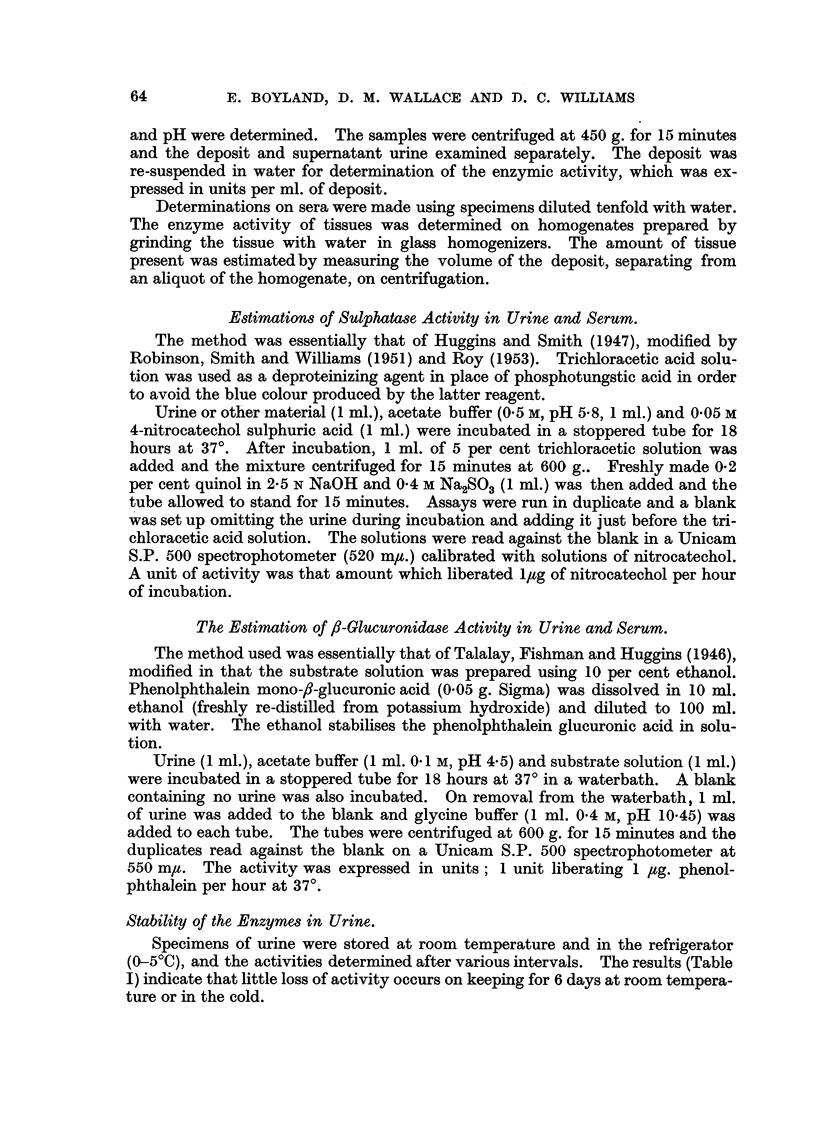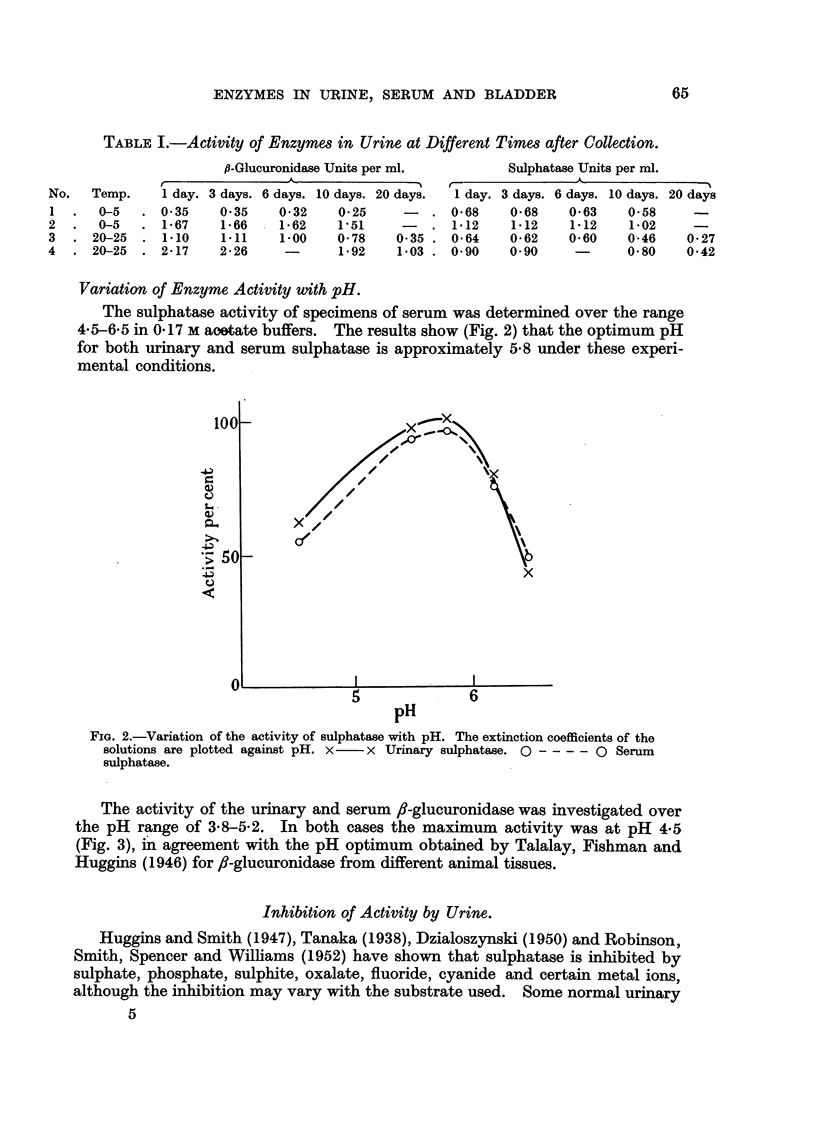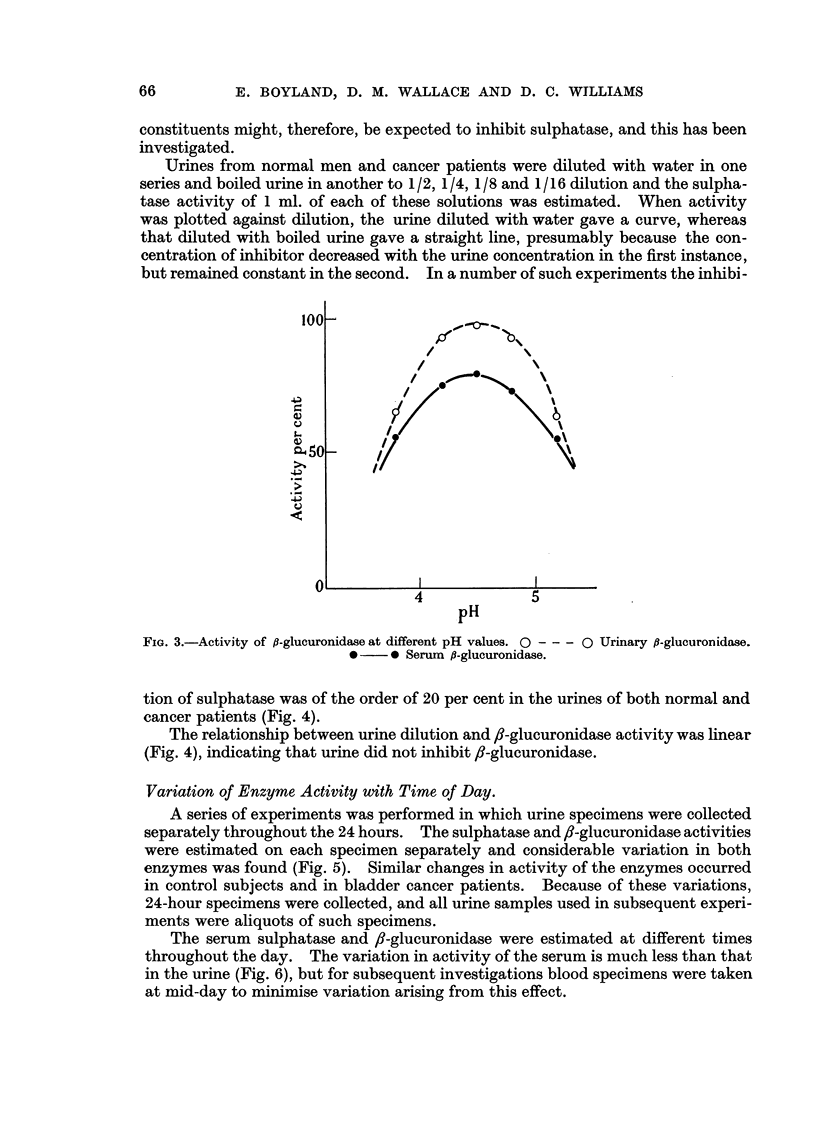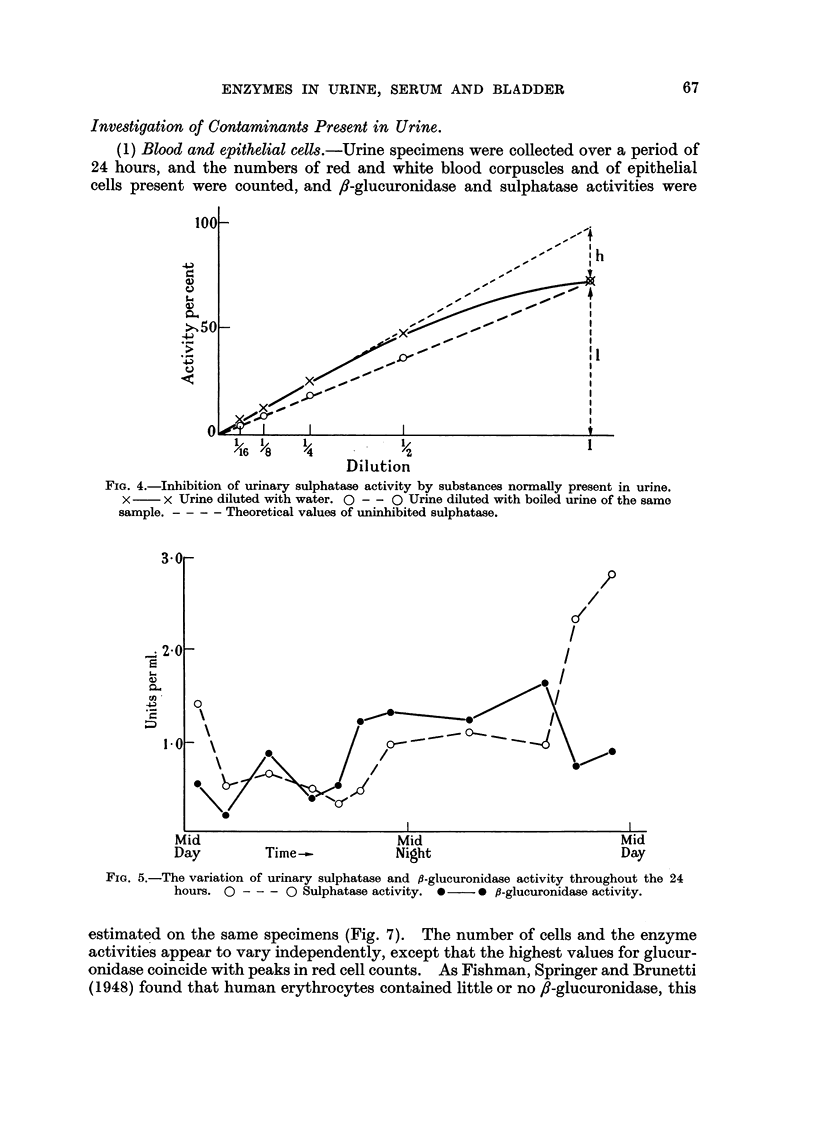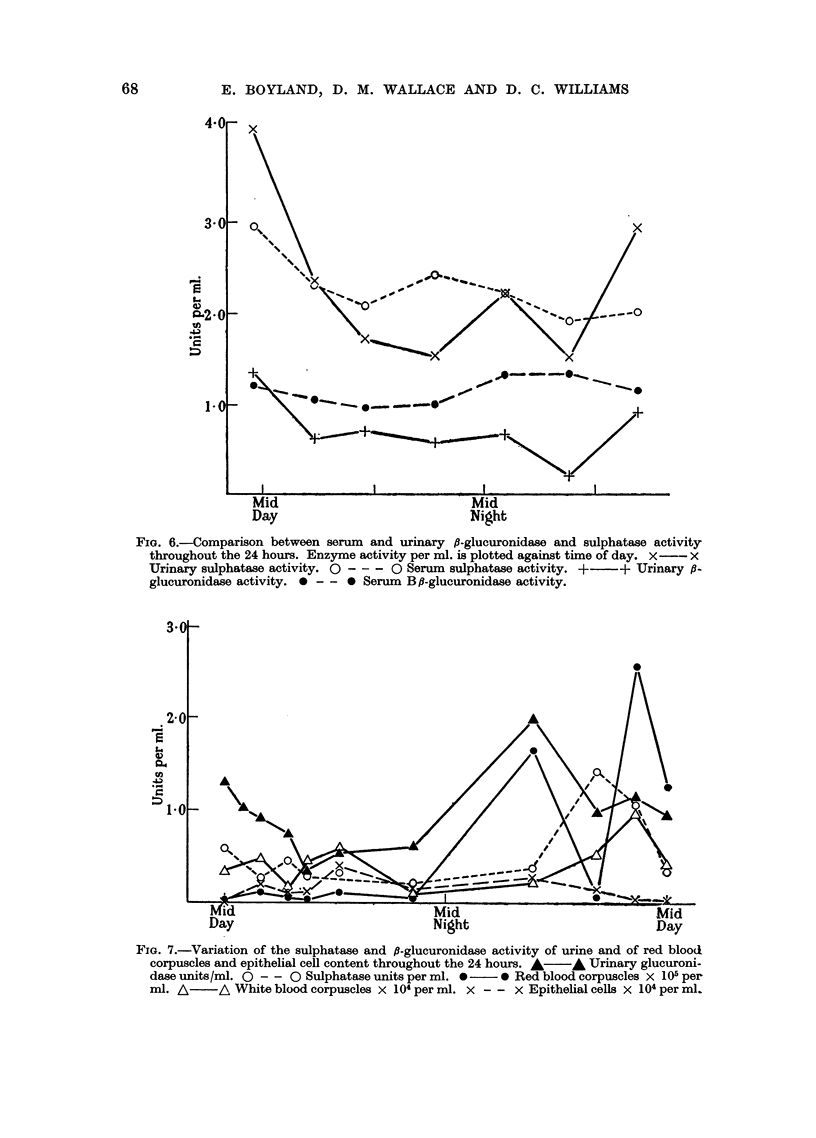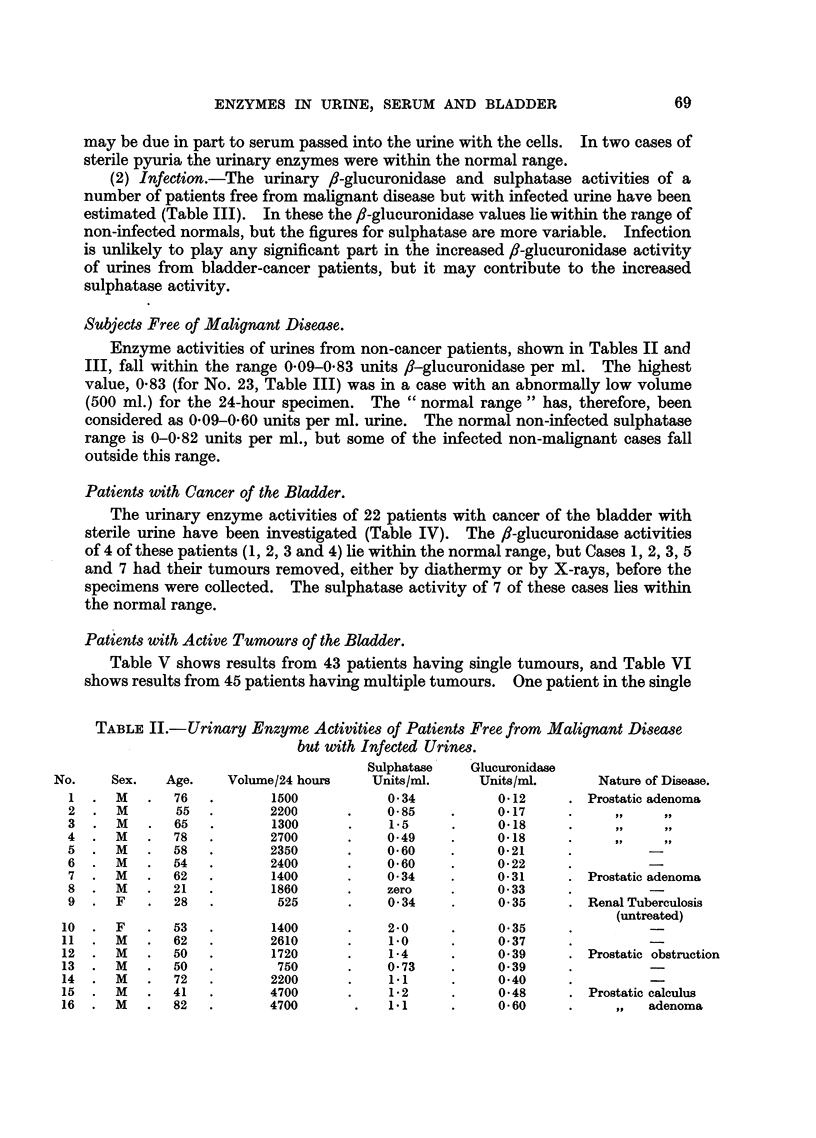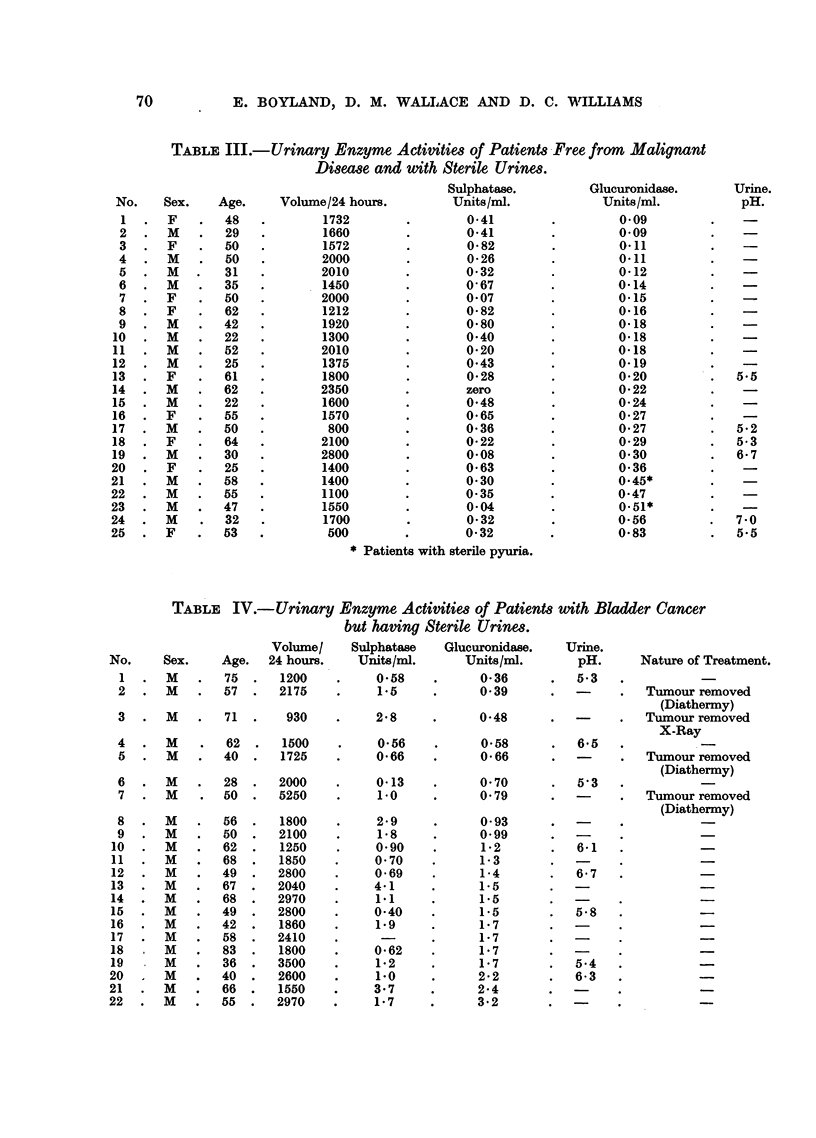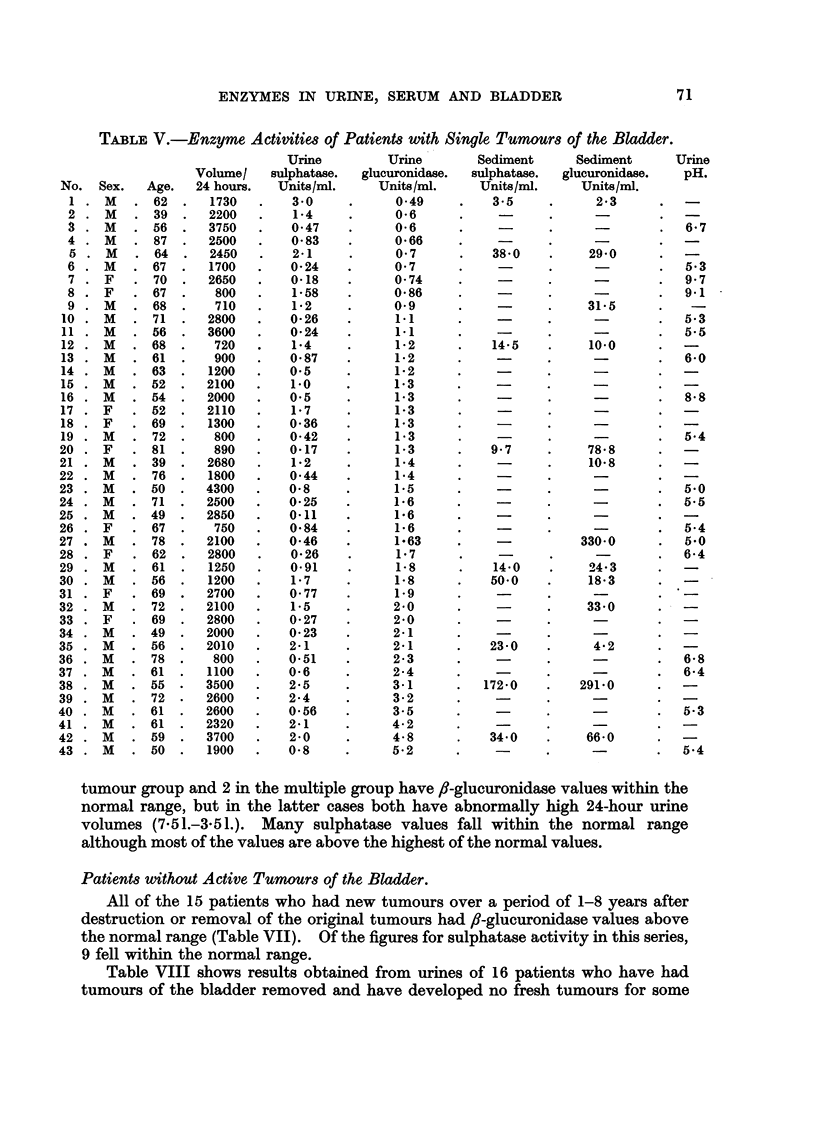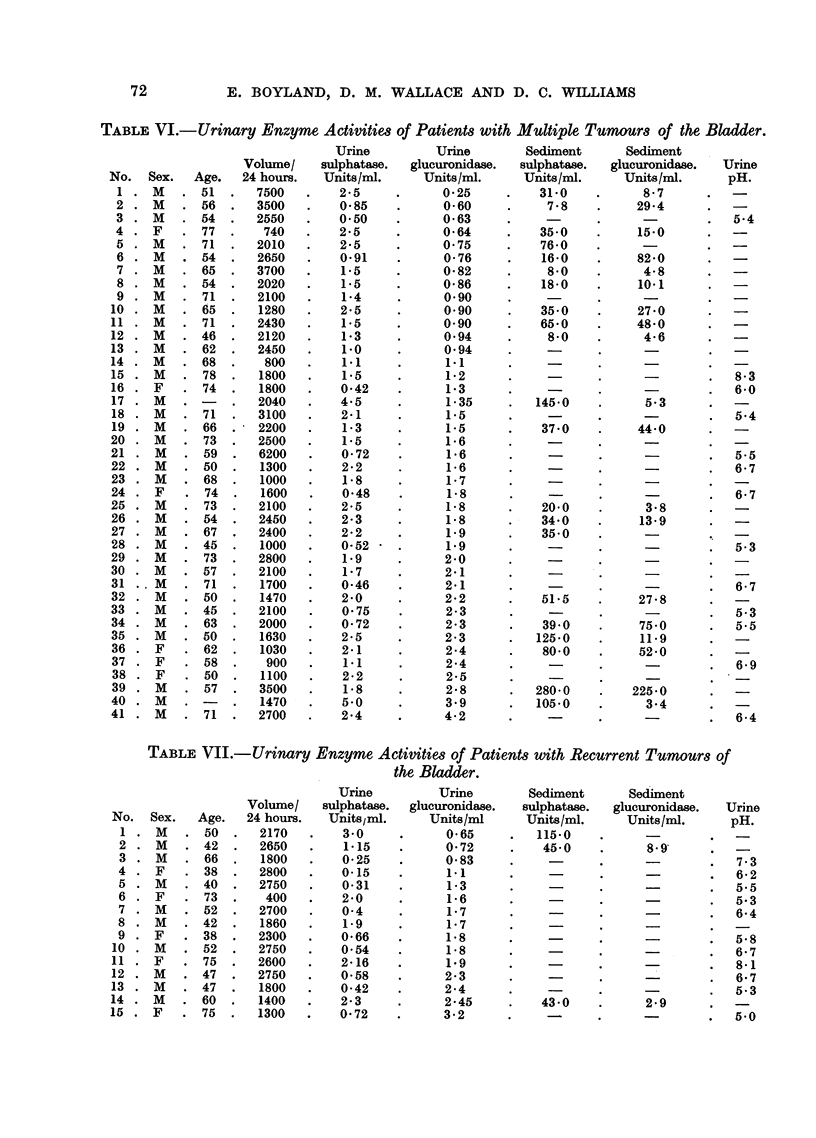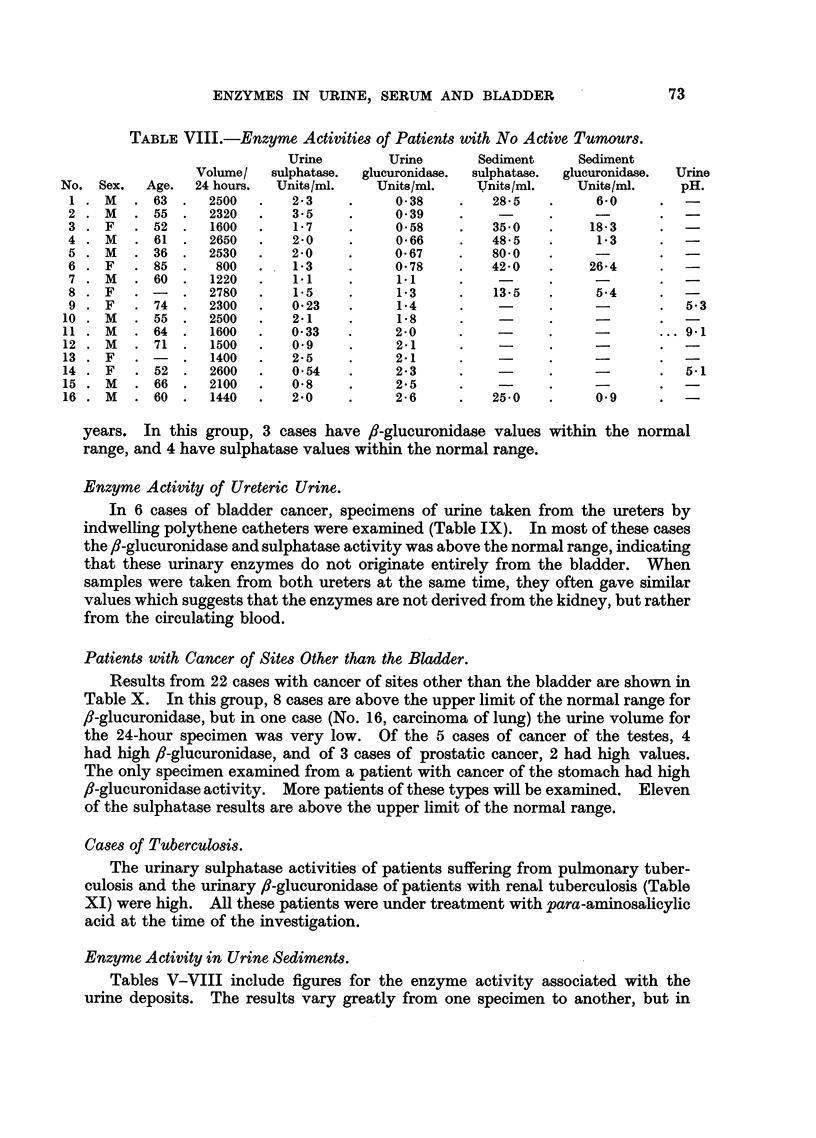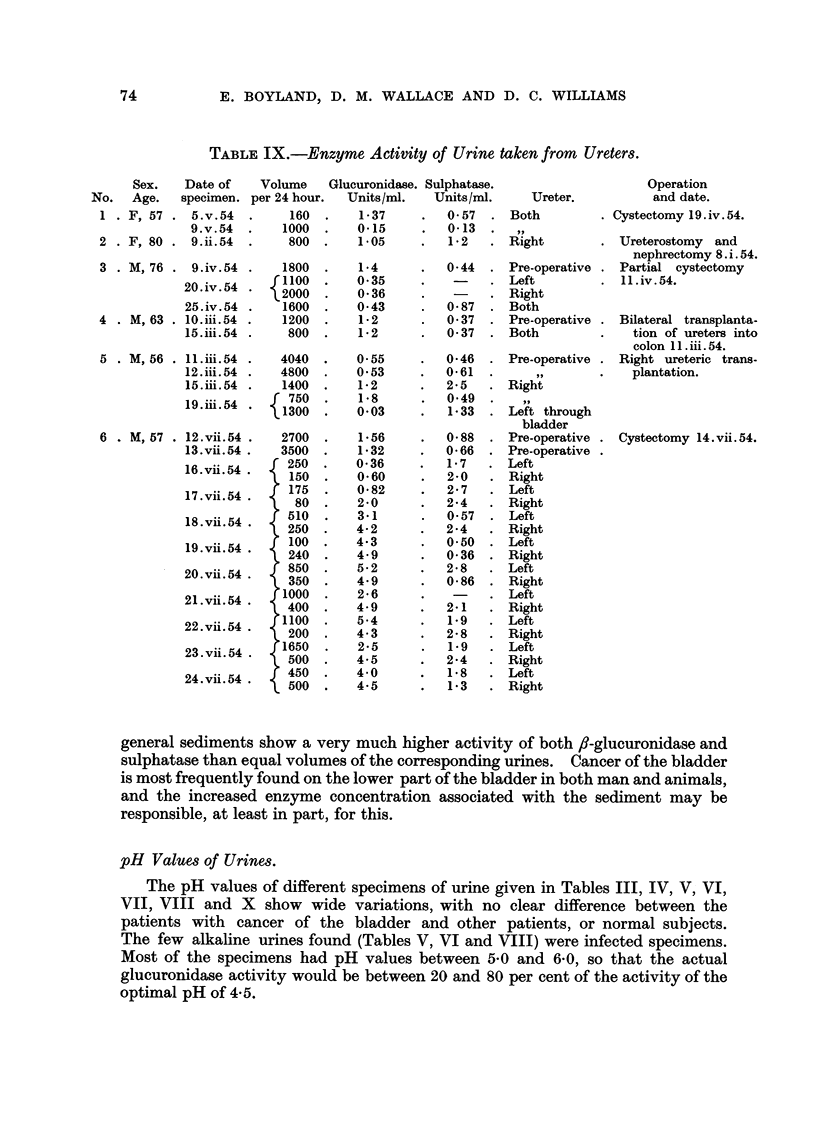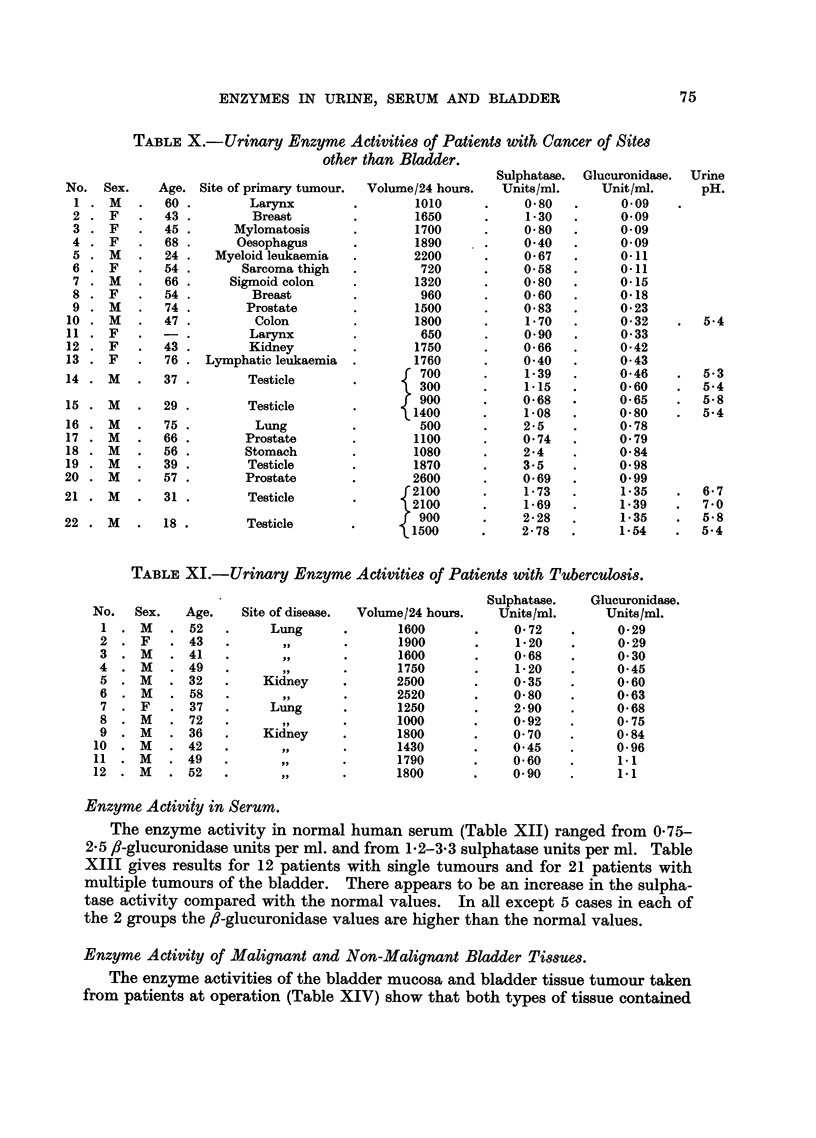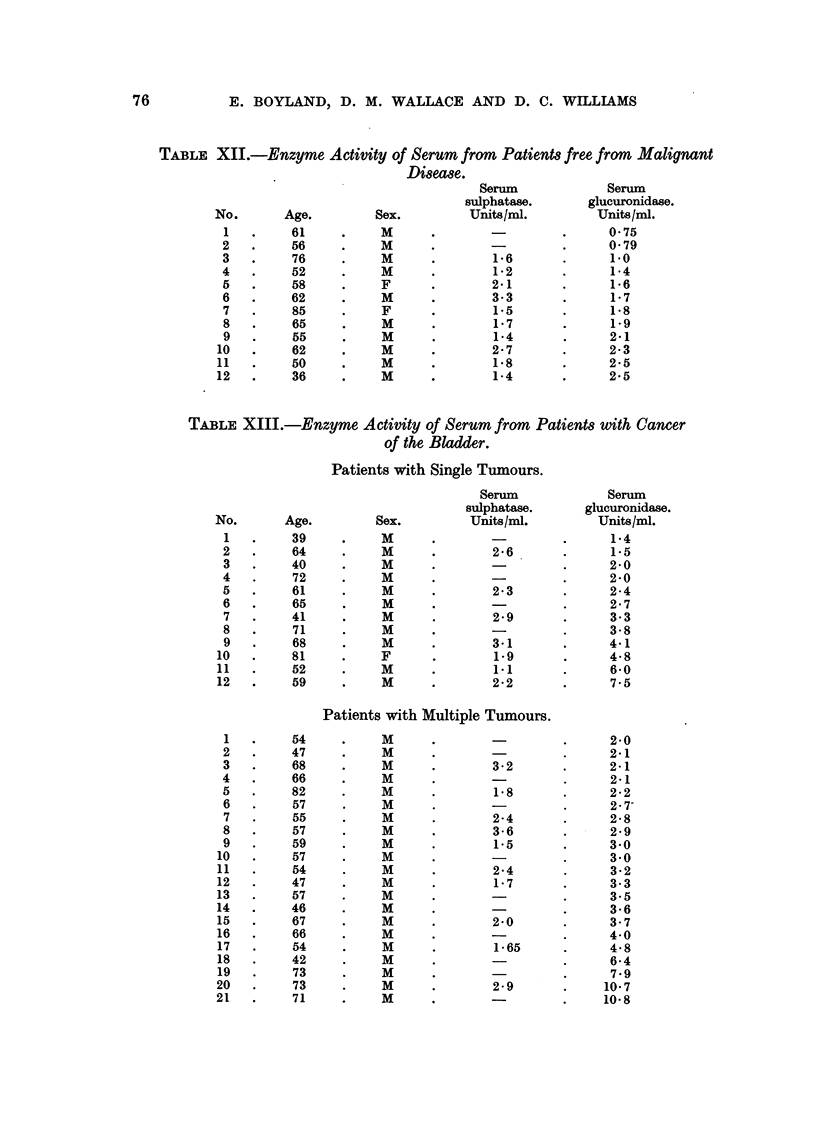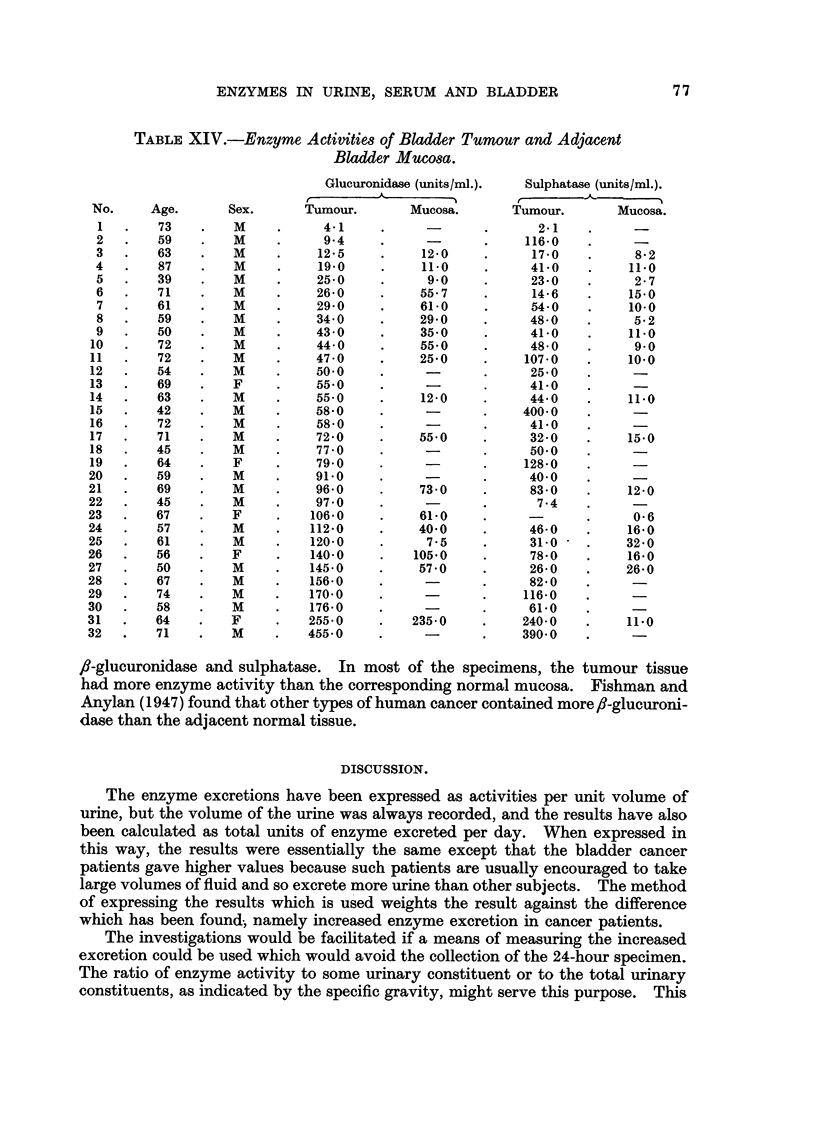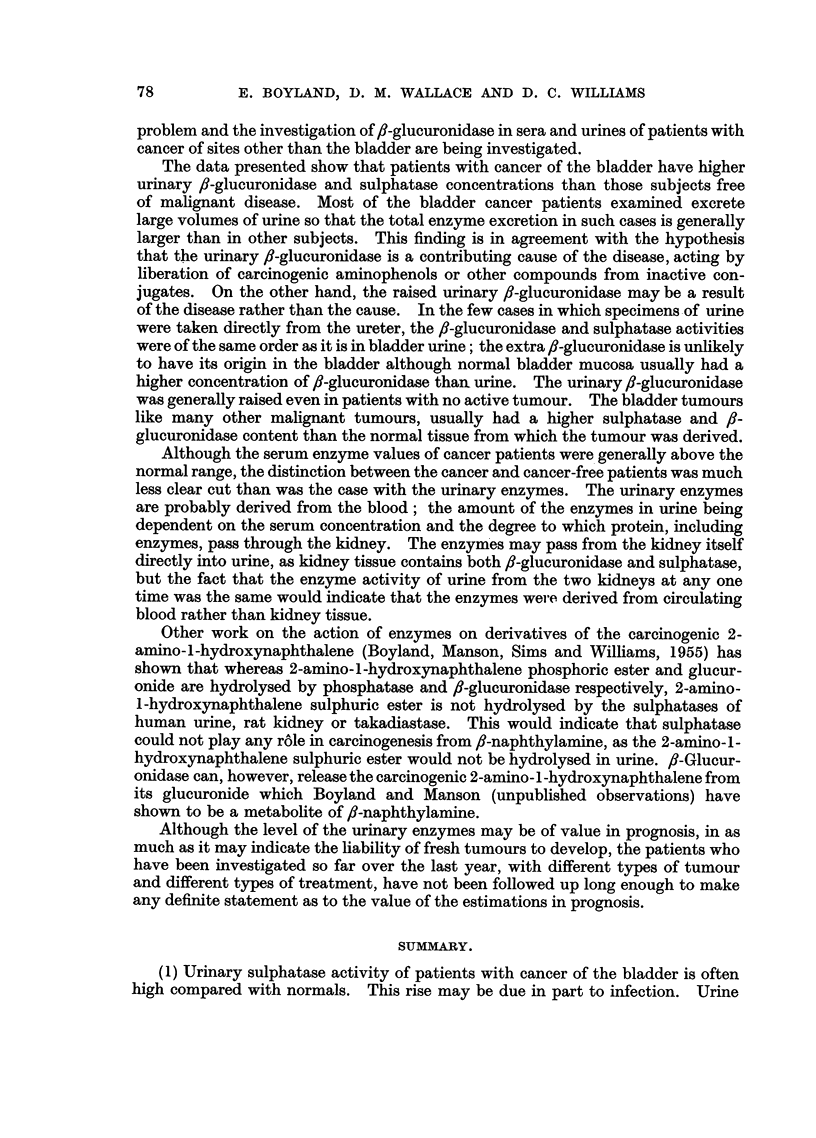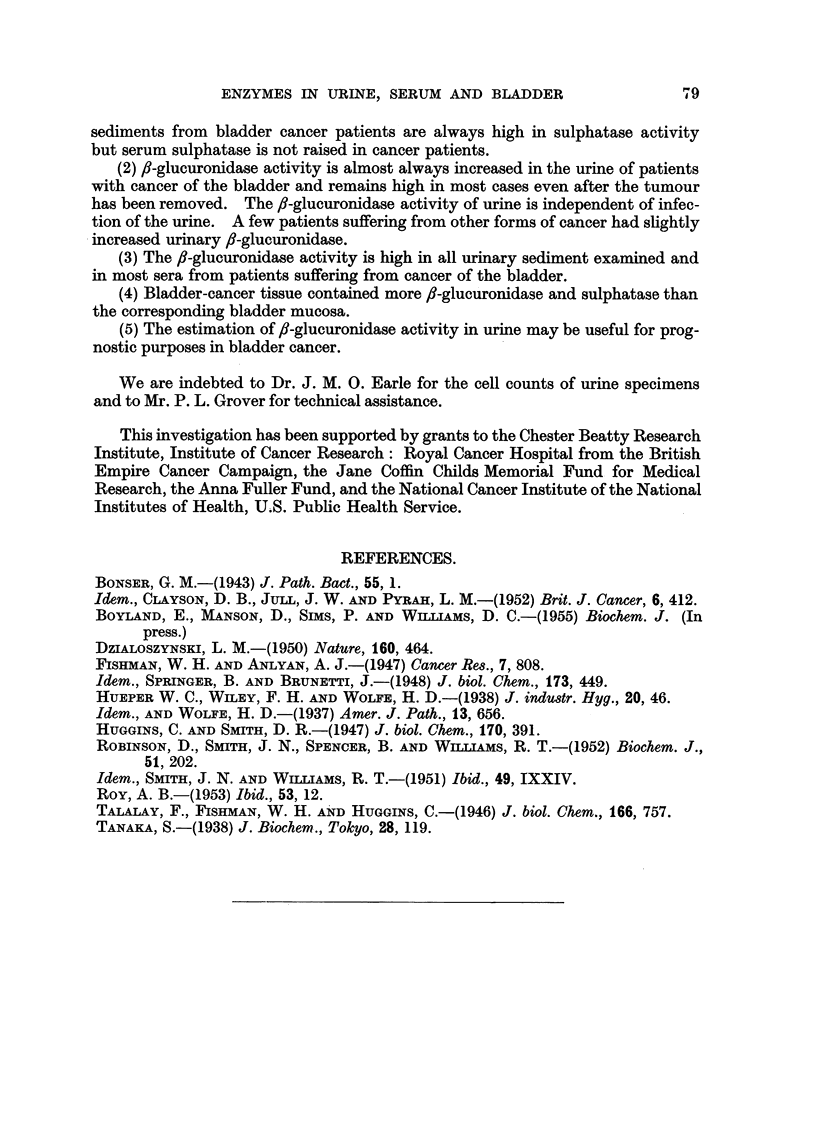# The Activity of the Enzymes Sulphatase and β-Glucuronidase in the Urine, Serum and Bladder Tissue

**DOI:** 10.1038/bjc.1955.6

**Published:** 1955-03

**Authors:** E. Boyland, D. M. Wallace, D. C. Williams


### THE ACTIVITY OF THE ENZYMES SULPHATASE AND β-GLUCURONIDASE IN THE URINE, SERUM AND BLADDER TISSUE.

E. BOYLAND, D. M. WALLACE AND D. C. WILLIAMS.

*From The Chester Beatty Research Institute ; Institute of Cancer Research ; Royal Cancer Hospital ; the Royal Marsden Hospital, London, S.W.3. ; and the Institute of Urology, London, W.C.2.*

Received for publication November 25, 1954.

THE fact that cancer of the bladder appears to be produced in men by external chemical agents used as dyestuff intermediates, and that bladder cancer can be produced in animals by a number of chemical substances, suggests that some bladder cancers in men, which are now considered to be spontaneous, are due to unidentified chemical carcinogens derived either from the environment or from the metabolic processes of the organism itself.

The incidence of cancer of the bladder is very high amongst men working in the chemical industry who have had contact with β-naphthylamine, α-naphthylamine or benzidine. Such tumours occurring in chemical workers are similar to those which occur in the general population not exposed to any known hazard.

Patients with papillary tumours of the bladder which have arisen spontaneously are liable to develop multiple tumours throughout the renal tract, either simultaneously or over a period of some years, in spite of any treatment that they have been given. These tumours occur in those parts of the renal tract where urine remains for some time, such as the lower end of the ureter, the pelvis of the kidney, the bladder, and, if present, a diverticulum. Tumours are rarely found in the upper third of the ureter, in the posterior urethra or the anterior urethra. In addition to these multiple tumours, areas of epithelial hyperplasia or even pre-malignant changes in the mucosa in such bladders are often seen. The distribution of these changes suggests that the mucosa has been exposed to a hyperplastic or carcinogenic agent present in the urine.

The symptomatology of some bladder tumours suggests that there may be a phase of irritation preceding the appearance of a tumour. A patient may be treated for an abacterial cystitis for a year or two before a tumour is recognised. The cause of this " cystitis " may be a chemical rather than a bacterial irritant.

Cases have been recorded where, following transplantation of the ureters, tumours appear to have regressed spontaneously, and diversion of the urine may have played a significant part in the regression. Such patients might have a metabolic pattern analogous to an " inborn error of metabolism " or other cause such as vitamin deficiency associated with excretion of a carcinogenic agent.

The investigations of Hueper and Wolfe (1937), of Hueper, Wiley and Wolfe (1938), those of Bonser (1943) and of Bonser, Clayson, Jull and Pyrah (1952) and others have shown that 2-amino-1-hydroxynaphthalene and some other *ortho*-aminophenols can induce bladder cancer in mice and in dogs. Among the substances present in human urine are two *ortho*-aminophenols, 3-hydroxykynurenine and 3-hydroxyanthranilic acid, which are known metabolites of tryptophan (Fig. 1). One of the chemical reactions involved in the production of these substances is similar to the conversion of $\beta$-naphthylamine to the carcinogenic 2-amino-1-hydroxynaphthalene.

FIG. 1.—The metabolisation of tryptophan.

Aminophenols are usually excreted as sulphuric esters or as glucuronides in the urine, and these compounds would be expected to be inactive as carcinogens. The effective aminophenol concentration in urine would therefore depend upon :

(1) The total concentration of *ortho*-aminophenols and their conjugates.

(2) The time during which the urine remained in the bladder during which the aminophenols could be liberated from conjugates by action of urinary enzymes.

(3) The activities of the enzymes $\beta$-glucuronidase and sulphatase which would liberate the free *ortho*-aminophenols from their conjugates. The enzyme activities will depend on the enzyme concentrations, the pH of the urine, and the concentrations of the enzyme inhibitors or activators.

In examining this hypothesis we propose to investigate :

(1) The carcinogenic activity of the *ortho*-aminophenols and other substances found in the urine of normal subjects and of patients with cancer of the bladder.

(2) The amounts of individual *ortho*-aminophenols and their conjugation products present in normal and pathological urines.

(3) The activities of certain hydrolysing enzymes in these urines.

The following is an account of the estimation of the activities of the enzymes sulphatase and $\beta$-glucuronidase in blood and urine of normal subjects and in blood serum and bladder tissues of patients suffering from malignant disease.

### EXPERIMENTAL.

Urine from hospital patients and normal individuals was collected in vessels containing small volumes of benzene as preservative. Individual specimens or 24 hour specimens were measured, and in the later investigations specific gravity and pH were determined. The samples were centrifuged at 450 g. for 15 minutes and the deposit and supernatant urine examined separately. The deposit was re-suspended in water for determination of the enzymic activity, which was expressed in units per ml. of deposit.

Determinations on sera were made using specimens diluted tenfold with water. The enzyme activity of tissues was determined on homogenates prepared by grinding the tissue with water in glass homogenizers. The amount of tissue present was estimated by measuring the volume of the deposit, separating from an aliquot of the homogenate, on centrifugation.

*Estimations of Sulphatase Activity in Urine and Serum.*

The method was essentially that of Huggins and Smith (1947), modified by Robinson, Smith and Williams (1951) and Roy (1953). Trichloracetic acid solution was used as a deproteinizing agent in place of phosphotungstic acid in order to avoid the blue colour produced by the latter reagent.

Urine or other material (1 ml.), acetate buffer (0·5 M, pH 5·8, 1 ml.) and 0·05 M 4-nitrocatechol sulphuric acid (1 ml.) were incubated in a stoppered tube for 18 hours at 37°. After incubation, 1 ml. of 5 per cent trichloracetic solution was added and the mixture centrifuged for 15 minutes at 600 g.. Freshly made 0·2 per cent quinol in 2·5 N NaOH and 0·4 M $Na_2SO_3$ (1 ml.) was then added and the tube allowed to stand for 15 minutes. Assays were run in duplicate and a blank was set up omitting the urine during incubation and adding it just before the trichloracetic acid solution. The solutions were read against the blank in a Unicam S.P. 500 spectrophotometer (520 m$\mu$.) calibrated with solutions of nitrocatechol. A unit of activity was that amount which liberated 1$\mu$g of nitrocatechol per hour of incubation.

*The Estimation of β-Glucuronidase Activity in Urine and Serum.*

The method used was essentially that of Talalay, Fishman and Huggins (1946), modified in that the substrate solution was prepared using 10 per cent ethanol. Phenolphthalein mono-β-glucuronic acid (0·05 g. Sigma) was dissolved in 10 ml. ethanol (freshly re-distilled from potassium hydroxide) and diluted to 100 ml. with water. The ethanol stabilises the phenolphthalein glucuronic acid in solution.

Urine (1 ml.), acetate buffer (1 ml. 0·1 M, pH 4·5) and substrate solution (1 ml.) were incubated in a stoppered tube for 18 hours at 37° in a waterbath. A blank containing no urine was also incubated. On removal from the waterbath, 1 ml. of urine was added to the blank and glycine buffer (1 ml. 0·4 M, pH 10·45) was added to each tube. The tubes were centrifuged at 600 g. for 15 minutes and the duplicates read against the blank on a Unicam S.P. 500 spectrophotometer at 550 m$\mu$. The activity was expressed in units ; 1 unit liberating 1 $\mu$g. phenolphthalein per hour at 37°.

*Stability of the Enzymes in Urine.*

Specimens of urine were stored at room temperature and in the refrigerator (0–5°C), and the activities determined after various intervals. The results (Table I) indicate that little loss of activity occurs on keeping for 6 days at room temperature or in the cold.

TABLE I.—*Activity of Enzymes in Urine at Different Times after Collection.*

| | | β-Glucuronidase Units per ml. | | | | | Sulphatase Units per ml. | | | | |
|---|---|---|---|---|---|---|---|---|---|---|---|
| No. | Temp. | 1 day. | 3 days. | 6 days. | 10 days. | 20 days. | 1 day. | 3 days. | 6 days. | 10 days. | 20 days |
| 1 | 0–5 | 0·35 | 0·35 | 0·32 | 0·25 | — | 0·68 | 0·68 | 0·63 | 0·58 | — |
| 2 | 0–5 | 1·67 | 1·66 | 1·62 | 1·51 | — | 1·12 | 1·12 | 1·12 | 1·02 | — |
| 3 | 20–25 | 1·10 | 1·11 | 1·00 | 0·78 | 0·35 | 0·64 | 0·62 | 0·60 | 0·46 | 0·27 |
| 4 | 20–25 | 2·17 | 2·26 | — | 1·92 | 1·03 | 0·90 | 0·90 | — | 0·80 | 0·42 |

*Variation of Enzyme Activity with pH.*

The sulphatase activity of specimens of serum was determined over the range 4·5–6·5 in 0·17 M acetate buffers. The results show (Fig. 2) that the optimum pH for both urinary and serum sulphatase is approximately 5·8 under these experimental conditions.

FIG. 2.—Variation of the activity of sulphatase with pH. The extinction coefficients of the solutions are plotted against pH. X——X Urinary sulphatase. O - - - - O Serum sulphatase.

The activity of the urinary and serum β-glucuronidase was investigated over the pH range of 3·8–5·2. In both cases the maximum activity was at pH 4·5 (Fig. 3), in agreement with the pH optimum obtained by Talalay, Fishman and Huggins (1946) for β-glucuronidase from different animal tissues.

*Inhibition of Activity by Urine.*

Huggins and Smith (1947), Tanaka (1938), Dzialoszynski (1950) and Robinson, Smith, Spencer and Williams (1952) have shown that sulphatase is inhibited by sulphate, phosphate, sulphite, oxalate, fluoride, cyanide and certain metal ions, although the inhibition may vary with the substrate used. Some normal urinary constituents might, therefore, be expected to inhibit sulphatase, and this has been investigated.

Urines from normal men and cancer patients were diluted with water in one series and boiled urine in another to 1/2, 1/4, 1/8 and 1/16 dilution and the sulphatase activity of 1 ml. of each of these solutions was estimated. When activity was plotted against dilution, the urine diluted with water gave a curve, whereas that diluted with boiled urine gave a straight line, presumably because the concentration of inhibitor decreased with the urine concentration in the first instance, but remained constant in the second. In a number of such experiments the inhibition of sulphatase was of the order of 20 per cent in the urines of both normal and cancer patients (Fig. 4).

FIG. 3.—Activity of β-glucuronidase at different pH values. ○ – – – ○ Urinary β-glucuronidase. ● —— ● Serum β-glucuronidase.

The relationship between urine dilution and β-glucuronidase activity was linear (Fig. 4), indicating that urine did not inhibit β-glucuronidase.

*Variation of Enzyme Activity with Time of Day.*

A series of experiments was performed in which urine specimens were collected separately throughout the 24 hours. The sulphatase and β-glucuronidase activities were estimated on each specimen separately and considerable variation in both enzymes was found (Fig. 5). Similar changes in activity of the enzymes occurred in control subjects and in bladder cancer patients. Because of these variations, 24-hour specimens were collected, and all urine samples used in subsequent experiments were aliquots of such specimens.

The serum sulphatase and β-glucuronidase were estimated at different times throughout the day. The variation in activity of the serum is much less than that in the urine (Fig. 6), but for subsequent investigations blood specimens were taken at mid-day to minimise variation arising from this effect.

*Investigation of Contaminants Present in Urine.*

(1) *Blood and epithelial cells.*—Urine specimens were collected over a period of 24 hours, and the numbers of red and white blood corpuscles and of epithelial cells present were counted, and $\beta$-glucuronidase and sulphatase activities were

Fig. 4.—Inhibition of urinary sulphatase activity by substances normally present in urine. ×——× Urine diluted with water. ○ – – ○ Urine diluted with boiled urine of the same sample. – – – – Theoretical values of uninhibited sulphatase.

Fig. 5.—The variation of urinary sulphatase and $\beta$-glucuronidase activity throughout the 24 hours. ○ – – – ○ Sulphatase activity. ●——● $\beta$-glucuronidase activity.

estimated on the same specimens (Fig. 7). The number of cells and the enzyme activities appear to vary independently, except that the highest values for glucuronidase coincide with peaks in red cell counts. As Fishman, Springer and Brunetti (1948) found that human erythrocytes contained little or no $\beta$-glucuronidase, this

FIG. 6.—Comparison between serum and urinary β-glucuronidase and sulphatase activity throughout the 24 hours. Enzyme activity per ml. is plotted against time of day. × —— × Urinary sulphatase activity. ○ - - - ○ Serum sulphatase activity. + —— + Urinary β-glucuronidase activity. ● - - ● Serum Bβ-glucuronidase activity.

FIG. 7.—Variation of the sulphatase and β-glucuronidase activity of urine and of red blood corpuscles and epithelial cell content throughout the 24 hours. ▲ —— ▲ Urinary glucuronidase units/ml. ○ - - ○ Sulphatase units per ml. ● —— ● Red blood corpuscles × $10^5$ per ml. △ —— △ White blood corpuscles × $10^4$ per ml. × - - × Epithelial cells × $10^4$ per ml.

may be due in part to serum passed into the urine with the cells. In two cases of sterile pyuria the urinary enzymes were within the normal range.

(2) *Infection*.—The urinary $\beta$-glucuronidase and sulphatase activities of a number of patients free from malignant disease but with infected urine have been estimated (Table III). In these the $\beta$-glucuronidase values lie within the range of non-infected normals, but the figures for sulphatase are more variable. Infection is unlikely to play any significant part in the increased $\beta$-glucuronidase activity of urines from bladder-cancer patients, but it may contribute to the increased sulphatase activity.

*Subjects Free of Malignant Disease.*

Enzyme activities of urines from non-cancer patients, shown in Tables II and III, fall within the range 0·09–0·83 units $\beta$-glucuronidase per ml. The highest value, 0·83 (for No. 23, Table III) was in a case with an abnormally low volume (500 ml.) for the 24-hour specimen. The " normal range " has, therefore, been considered as 0·09–0·60 units per ml. urine. The normal non-infected sulphatase range is 0–0·82 units per ml., but some of the infected non-malignant cases fall outside this range.

*Patients with Cancer of the Bladder.*

The urinary enzyme activities of 22 patients with cancer of the bladder with sterile urine have been investigated (Table IV). The $\beta$-glucuronidase activities of 4 of these patients (1, 2, 3 and 4) lie within the normal range, but Cases 1, 2, 3, 5 and 7 had their tumours removed, either by diathermy or by X-rays, before the specimens were collected. The sulphatase activity of 7 of these cases lies within the normal range.

*Patients with Active Tumours of the Bladder.*

Table V shows results from 43 patients having single tumours, and Table VI shows results from 45 patients having multiple tumours. One patient in the single

TABLE II.—*Urinary Enzyme Activities of Patients Free from Malignant Disease but with Infected Urines.*

| No. | Sex. | Age. | Volume/24 hours | Sulphatase Units/ml. | Glucuronidase Units/ml. | Nature of Disease. |
|---|---|---|---|---|---|---|
| 1 | M | 76 | 1500 | 0·34 | 0·12 | Prostatic adenoma |
| 2 | M | 55 | 2200 | 0·85 | 0·17 | ,, ,, |
| 3 | M | 65 | 1300 | 1·5 | 0·18 | ,, ,, |
| 4 | M | 78 | 2700 | 0·49 | 0·18 | ,, ,, |
| 5 | M | 58 | 2350 | 0·60 | 0·21 | — |
| 6 | M | 54 | 2400 | 0·60 | 0·22 | — |
| 7 | M | 62 | 1400 | 0·34 | 0·31 | Prostatic adenoma |
| 8 | M | 21 | 1860 | zero | 0·33 | — |
| 9 | F | 28 | 525 | 0·34 | 0·35 | Renal Tuberculosis (untreated) |
| 10 | F | 53 | 1400 | 2·0 | 0·35 | — |
| 11 | M | 62 | 2610 | 1·0 | 0·37 | — |
| 12 | M | 50 | 1720 | 1·4 | 0·39 | Prostatic obstruction |
| 13 | M | 50 | 750 | 0·73 | 0·39 | — |
| 14 | M | 72 | 2200 | 1·1 | 0·40 | — |
| 15 | M | 41 | 4700 | 1·2 | 0·48 | Prostatic calculus |
| 16 | M | 82 | 4700 | 1·1 | 0·60 | ,, adenoma |

TABLE III.—*Urinary Enzyme Activities of Patients Free from Malignant Disease and with Sterile Urines.*

| No. | Sex. | Age. | Volume/24 hours. | Sulphatase. Units/ml. | Glucuronidase. Units/ml. | Urine. pH. |
|---|---|---|---|---|---|---|
| 1 | F | 48 | 1732 | 0·41 | 0·09 | — |
| 2 | M | 29 | 1660 | 0·41 | 0·09 | — |
| 3 | F | 50 | 1572 | 0·82 | 0·11 | — |
| 4 | M | 50 | 2000 | 0·26 | 0·11 | — |
| 5 | M | 31 | 2010 | 0·32 | 0·12 | — |
| 6 | M | 35 | 1450 | 0·67 | 0·14 | — |
| 7 | F | 50 | 2000 | 0·07 | 0·15 | — |
| 8 | F | 62 | 1212 | 0·82 | 0·16 | — |
| 9 | M | 42 | 1920 | 0·80 | 0·18 | — |
| 10 | M | 22 | 1300 | 0·40 | 0·18 | — |
| 11 | M | 52 | 2010 | 0·20 | 0·18 | — |
| 12 | M | 25 | 1375 | 0·43 | 0·19 | — |
| 13 | F | 61 | 1800 | 0·28 | 0·20 | 5·5 |
| 14 | M | 62 | 2350 | zero | 0·22 | — |
| 15 | M | 22 | 1600 | 0·48 | 0·24 | — |
| 16 | F | 55 | 1570 | 0·65 | 0·27 | — |
| 17 | M | 50 | 800 | 0·36 | 0·27 | 5·2 |
| 18 | F | 64 | 2100 | 0·22 | 0·29 | 5·3 |
| 19 | M | 30 | 2800 | 0·08 | 0·30 | 6·7 |
| 20 | F | 25 | 1400 | 0·63 | 0·36 | — |
| 21 | M | 58 | 1400 | 0·30 | 0·45* | — |
| 22 | M | 55 | 1100 | 0·35 | 0·47 | — |
| 23 | M | 47 | 1550 | 0·04 | 0·51* | — |
| 24 | M | 32 | 1700 | 0·32 | 0·56 | 7·0 |
| 25 | F | 53 | 500 | 0·32 | 0·83 | 5·5 |

* Patients with sterile pyuria.

TABLE IV.—*Urinary Enzyme Activities of Patients with Bladder Cancer but having Sterile Urines.*

| No. | Sex. | Age. | Volume/ 24 hours. | Sulphatase Units/ml. | Glucuronidase. Units/ml. | Urine. pH. | Nature of Treatment. |
|---|---|---|---|---|---|---|---|
| 1 | M | 75 | 1200 | 0·58 | 0·36 | 5·3 | — |
| 2 | M | 57 | 2175 | 1·5 | 0·39 | — | Tumour removed (Diathermy) |
| 3 | M | 71 | 930 | 2·8 | 0·48 | — | Tumour removed X-Ray |
| 4 | M | 62 | 1500 | 0·56 | 0·58 | 6·5 | — |
| 5 | M | 40 | 1725 | 0·66 | 0·66 | — | Tumour removed (Diathermy) |
| 6 | M | 28 | 2000 | 0·13 | 0·70 | 5·3 | — |
| 7 | M | 50 | 5250 | 1·0 | 0·79 | — | Tumour removed (Diathermy) |
| 8 | M | 56 | 1800 | 2·9 | 0·93 | — | — |
| 9 | M | 50 | 2100 | 1·8 | 0·99 | — | — |
| 10 | M | 62 | 1250 | 0·90 | 1·2 | 6·1 | — |
| 11 | M | 68 | 1850 | 0·70 | 1·3 | — | — |
| 12 | M | 49 | 2800 | 0·69 | 1·4 | 6·7 | — |
| 13 | M | 67 | 2040 | 4·1 | 1·5 | — | — |
| 14 | M | 68 | 2970 | 1·1 | 1·5 | — | — |
| 15 | M | 49 | 2800 | 0·40 | 1·5 | 5·8 | — |
| 16 | M | 42 | 1860 | 1·9 | 1·7 | — | — |
| 17 | M | 58 | 2410 | — | 1·7 | — | — |
| 18 | M | 83 | 1800 | 0·62 | 1·7 | — | — |
| 19 | M | 36 | 3500 | 1·2 | 1·7 | 5·4 | — |
| 20 | M | 40 | 2600 | 1·0 | 2·2 | 6·3 | — |
| 21 | M | 66 | 1550 | 3·7 | 2·4 | — | — |
| 22 | M | 55 | 2970 | 1·7 | 3·2 | — | — |

TABLE V.—*Enzyme Activities of Patients with Single Tumours of the Bladder.*

| No. | Sex. | Age. | Volume/ 24 hours. | Urine sulphatase. Units/ml. | Urine glucuronidase. Units/ml. | Sediment sulphatase. Units/ml. | Sediment glucuronidase. Units/ml. | Urine pH. |
|---|---|---|---|---|---|---|---|---|
| 1 | M | 62 | 1730 | 3·0 | 0·49 | 3·5 | 2·3 | — |
| 2 | M | 39 | 2200 | 1·4 | 0·6 | — | — | — |
| 3 | M | 56 | 3750 | 0·47 | 0·6 | — | — | 6·7 |
| 4 | M | 87 | 2500 | 0·83 | 0·66 | — | — | — |
| 5 | M | 64 | 2450 | 2·1 | 0·7 | 38·0 | 29·0 | — |
| 6 | M | 67 | 1700 | 0·24 | 0·7 | — | — | 5·3 |
| 7 | F | 70 | 2650 | 0·18 | 0·74 | — | — | 9·7 |
| 8 | F | 67 | 800 | 1·58 | 0·86 | — | — | 9·1 |
| 9 | M | 68 | 710 | 1·2 | 0·9 | — | 31·5 | — |
| 10 | M | 71 | 2800 | 0·26 | 1·1 | — | — | 5·3 |
| 11 | M | 56 | 3600 | 0·24 | 1·1 | — | — | 5·5 |
| 12 | M | 68 | 720 | 1·4 | 1·2 | 14·5 | 10·0 | — |
| 13 | M | 61 | 900 | 0·87 | 1·2 | — | — | 6·0 |
| 14 | M | 63 | 1200 | 0·5 | 1·2 | — | — | — |
| 15 | M | 52 | 2100 | 1·0 | 1·3 | — | — | — |
| 16 | M | 54 | 2000 | 0·5 | 1·3 | — | — | 8·8 |
| 17 | F | 52 | 2110 | 1·7 | 1·3 | — | — | — |
| 18 | F | 69 | 1300 | 0·36 | 1·3 | — | — | — |
| 19 | M | 72 | 800 | 0·42 | 1·3 | — | — | 5·4 |
| 20 | F | 81 | 890 | 0·17 | 1·3 | 9·7 | 78·8 | — |
| 21 | M | 39 | 2680 | 1·2 | 1·4 | — | 10·8 | — |
| 22 | M | 76 | 1800 | 0·44 | 1·4 | — | — | — |
| 23 | M | 50 | 4300 | 0·8 | 1·5 | — | — | 5·0 |
| 24 | M | 71 | 2500 | 0·25 | 1·6 | — | — | 5·5 |
| 25 | M | 49 | 2850 | 0·11 | 1·6 | — | — | — |
| 26 | F | 67 | 750 | 0·84 | 1·6 | — | — | 5·4 |
| 27 | M | 78 | 2100 | 0·46 | 1·63 | — | 330·0 | 5·0 |
| 28 | F | 62 | 2800 | 0·26 | 1·7 | — | — | 6·4 |
| 29 | M | 61 | 1250 | 0·91 | 1·8 | 14·0 | 24·3 | — |
| 30 | M | 56 | 1200 | 1·7 | 1·8 | 50·0 | 18·3 | — |
| 31 | F | 69 | 2700 | 0·77 | 1·9 | — | — | — |
| 32 | M | 72 | 2100 | 1·5 | 2·0 | — | 33·0 | — |
| 33 | F | 69 | 2800 | 0·27 | 2·0 | — | — | — |
| 34 | M | 49 | 2000 | 0·23 | 2·1 | — | — | — |
| 35 | M | 56 | 2010 | 2·1 | 2·1 | 23·0 | 4·2 | — |
| 36 | M | 78 | 800 | 0·51 | 2·3 | — | — | 6·8 |
| 37 | M | 61 | 1100 | 0·6 | 2·4 | — | — | 6·4 |
| 38 | M | 55 | 3500 | 2·5 | 3·1 | 172·0 | 291·0 | — |
| 39 | M | 72 | 2600 | 2·4 | 3·2 | — | — | — |
| 40 | M | 61 | 2600 | 0·56 | 3·5 | — | — | 5·3 |
| 41 | M | 61 | 2320 | 2·1 | 4·2 | — | — | — |
| 42 | M | 59 | 3700 | 2·0 | 4·8 | 34·0 | 66·0 | — |
| 43 | M | 50 | 1900 | 0·8 | 5·2 | — | — | 5·4 |

tumour group and 2 in the multiple group have $\beta$-glucuronidase values within the normal range, but in the latter cases both have abnormally high 24-hour urine volumes (7·5 l.–3·5 l.). Many sulphatase values fall within the normal range although most of the values are above the highest of the normal values.

*Patients without Active Tumours of the Bladder.*

All of the 15 patients who had new tumours over a period of 1–8 years after destruction or removal of the original tumours had $\beta$-glucuronidase values above the normal range (Table VII). Of the figures for sulphatase activity in this series, 9 fell within the normal range.

Table VIII shows results obtained from urines of 16 patients who have had tumours of the bladder removed and have developed no fresh tumours for some

TABLE VI.—*Urinary Enzyme Activities of Patients with Multiple Tumours of the Bladder.*

| No. | Sex. | Age. | Volume/ 24 hours. | Urine sulphatase. Units/ml. | Urine glucuronidase. Units/ml. | Sediment sulphatase. Units/ml. | Sediment glucuronidase. Units/ml. | Urine pH. |
|---|---|---|---|---|---|---|---|---|
| 1 | M | 51 | 7500 | 2·5 | 0·25 | 31·0 | 8·7 | — |
| 2 | M | 56 | 3500 | 0·85 | 0·60 | 7·8 | 29·4 | — |
| 3 | M | 54 | 2550 | 0·50 | 0·63 | — | — | 5·4 |
| 4 | F | 77 | 740 | 2·5 | 0·64 | 35·0 | 15·0 | — |
| 5 | M | 71 | 2010 | 2·5 | 0·75 | 76·0 | — | — |
| 6 | M | 54 | 2650 | 0·91 | 0·76 | 16·0 | 82·0 | — |
| 7 | M | 65 | 3700 | 1·5 | 0·82 | 8·0 | 4·8 | — |
| 8 | M | 54 | 2020 | 1·5 | 0·86 | 18·0 | 10·1 | — |
| 9 | M | 71 | 2100 | 1·4 | 0·90 | — | — | — |
| 10 | M | 65 | 1280 | 2·5 | 0·90 | 35·0 | 27·0 | — |
| 11 | M | 71 | 2430 | 1·5 | 0·90 | 65·0 | 48·0 | — |
| 12 | M | 46 | 2120 | 1·3 | 0·94 | 8·0 | 4·6 | — |
| 13 | M | 62 | 2450 | 1·0 | 0·94 | — | — | — |
| 14 | M | 68 | 800 | 1·1 | 1·1 | — | — | — |
| 15 | M | 78 | 1800 | 1·5 | 1·2 | — | — | 8·3 |
| 16 | F | 74 | 1800 | 0·42 | 1·3 | — | — | 6·0 |
| 17 | M | — | 2040 | 4·5 | 1·35 | 145·0 | 5·3 | — |
| 18 | M | 71 | 3100 | 2·1 | 1·5 | — | — | 5·4 |
| 19 | M | 66 | 2200 | 1·3 | 1·5 | 37·0 | 44·0 | — |
| 20 | M | 73 | 2500 | 1·5 | 1·6 | — | — | — |
| 21 | M | 59 | 6200 | 0·72 | 1·6 | — | — | 5·5 |
| 22 | M | 50 | 1300 | 2·2 | 1·6 | — | — | 6·7 |
| 23 | M | 68 | 1000 | 1·8 | 1·7 | — | — | — |
| 24 | F | 74 | 1600 | 0·48 | 1·8 | — | — | 6·7 |
| 25 | M | 73 | 2100 | 2·5 | 1·8 | 20·0 | 3·8 | — |
| 26 | M | 54 | 2450 | 2·3 | 1·8 | 34·0 | 13·9 | — |
| 27 | M | 67 | 2400 | 2·2 | 1·9 | 35·0 | — | — |
| 28 | M | 45 | 1000 | 0·52 | 1·9 | — | — | 5·3 |
| 29 | M | 73 | 2800 | 1·9 | 2·0 | — | — | — |
| 30 | M | 57 | 2100 | 1·7 | 2·1 | — | — | — |
| 31 | M | 71 | 1700 | 0·46 | 2·1 | — | — | 6·7 |
| 32 | M | 50 | 1470 | 2·0 | 2·2 | 51·5 | 27·8 | — |
| 33 | M | 45 | 2100 | 0·75 | 2·3 | — | — | 5·3 |
| 34 | M | 63 | 2000 | 0·72 | 2·3 | 39·0 | 75·0 | 5·5 |
| 35 | M | 50 | 1630 | 2·5 | 2·3 | 125·0 | 11·9 | — |
| 36 | F | 62 | 1030 | 2·1 | 2·4 | 80·0 | 52·0 | — |
| 37 | F | 58 | 900 | 1·1 | 2·4 | — | — | 6·9 |
| 38 | F | 50 | 1100 | 2·2 | 2·5 | — | — | — |
| 39 | M | 57 | 3500 | 1·8 | 2·8 | 280·0 | 225·0 | — |
| 40 | M | — | 1470 | 5·0 | 3·9 | 105·0 | 3·4 | — |
| 41 | M | 71 | 2700 | 2·4 | 4·2 | — | — | 6·4 |

TABLE VII.—*Urinary Enzyme Activities of Patients with Recurrent Tumours of the Bladder.*

| No. | Sex. | Age. | Volume/ 24 hours. | Urine sulphatase. Units/ml. | Urine glucuronidase. Units/ml | Sediment sulphatase. Units/ml. | Sediment glucuronidase. Units/ml. | Urine pH. |
|---|---|---|---|---|---|---|---|---|
| 1 | M | 50 | 2170 | 3·0 | 0·65 | 115·0 | — | — |
| 2 | M | 42 | 2650 | 1·15 | 0·72 | 45·0 | 8·9 | — |
| 3 | M | 66 | 1800 | 0·25 | 0·83 | — | — | 7·3 |
| 4 | F | 38 | 2800 | 0·15 | 1·1 | — | — | 6·2 |
| 5 | M | 40 | 2750 | 0·31 | 1·3 | — | — | 5·5 |
| 6 | F | 73 | 400 | 2·0 | 1·6 | — | — | 5·3 |
| 7 | M | 52 | 2700 | 0·4 | 1·7 | — | — | 6·4 |
| 8 | M | 42 | 1860 | 1·9 | 1·7 | — | — | — |
| 9 | F | 38 | 2300 | 0·66 | 1·8 | — | — | 5·8 |
| 10 | M | 52 | 2750 | 0·54 | 1·8 | — | — | 6·7 |
| 11 | F | 75 | 2600 | 2·16 | 1·9 | — | — | 8·1 |
| 12 | M | 47 | 2750 | 0·58 | 2·3 | — | — | 6·7 |
| 13 | M | 47 | 1800 | 0·42 | 2·4 | — | — | 5·3 |
| 14 | M | 60 | 1400 | 2·3 | 2·45 | 43·0 | 2·9 | — |
| 15 | F | 75 | 1300 | 0·72 | 3·2 | — | — | 5·0 |

TABLE VIII.—*Enzyme Activities of Patients with No Active Tumours.*

| No. | Sex. | Age. | Volume/ 24 hours. | Urine sulphatase. Units/ml. | Urine glucuronidase. Units/ml. | Sediment sulphatase. Units/ml. | Sediment glucuronidase. Units/ml. | Urine pH. |
|---|---|---|---|---|---|---|---|---|
| 1 | M | 63 | 2500 | 2·3 | 0·38 | 28·5 | 6·0 | — |
| 2 | M | 55 | 2320 | 3·5 | 0·39 | — | — | — |
| 3 | F | 52 | 1600 | 1·7 | 0·58 | 35·0 | 18·3 | — |
| 4 | M | 61 | 2650 | 2·0 | 0·66 | 48·5 | 1·3 | — |
| 5 | M | 36 | 2530 | 2·0 | 0·67 | 80·0 | — | — |
| 6 | F | 85 | 800 | 1·3 | 0·78 | 42·0 | 26·4 | — |
| 7 | M | 60 | 1220 | 1·1 | 1·1 | — | — | — |
| 8 | F | — | 2780 | 1·5 | 1·3 | 13·5 | 5·4 | — |
| 9 | F | 74 | 2300 | 0·23 | 1·4 | — | — | 5·3 |
| 10 | M | 55 | 2500 | 2·1 | 1·8 | — | — | — |
| 11 | M | 64 | 1600 | 0·33 | 2·0 | — | — | 9·1 |
| 12 | M | 71 | 1500 | 0·9 | 2·1 | — | — | — |
| 13 | F | — | 1400 | 2·5 | 2·1 | — | — | — |
| 14 | F | 52 | 2600 | 0·54 | 2·3 | — | — | 5·1 |
| 15 | M | 66 | 2100 | 0·8 | 2·5 | — | — | — |
| 16 | M | 60 | 1440 | 2·0 | 2·6 | 25·0 | 0·9 | — |

years. In this group, 3 cases have $\beta$-glucuronidase values within the normal range, and 4 have sulphatase values within the normal range.

*Enzyme Activity of Ureteric Urine.*

In 6 cases of bladder cancer, specimens of urine taken from the ureters by indwelling polythene catheters were examined (Table IX). In most of these cases the $\beta$-glucuronidase and sulphatase activity was above the normal range, indicating that these urinary enzymes do not originate entirely from the bladder. When samples were taken from both ureters at the same time, they often gave similar values which suggests that the enzymes are not derived from the kidney, but rather from the circulating blood.

*Patients with Cancer of Sites Other than the Bladder.*

Results from 22 cases with cancer of sites other than the bladder are shown in Table X. In this group, 8 cases are above the upper limit of the normal range for $\beta$-glucuronidase, but in one case (No. 16, carcinoma of lung) the urine volume for the 24-hour specimen was very low. Of the 5 cases of cancer of the testes, 4 had high $\beta$-glucuronidase, and of 3 cases of prostatic cancer, 2 had high values. The only specimen examined from a patient with cancer of the stomach had high $\beta$-glucuronidase activity. More patients of these types will be examined. Eleven of the sulphatase results are above the upper limit of the normal range.

*Cases of Tuberculosis.*

The urinary sulphatase activities of patients suffering from pulmonary tuberculosis and the urinary $\beta$-glucuronidase of patients with renal tuberculosis (Table XI) were high. All these patients were under treatment with *para*-aminosalicylic acid at the time of the investigation.

*Enzyme Activity in Urine Sediments.*

Tables V–VIII include figures for the enzyme activity associated with the urine deposits. The results vary greatly from one specimen to another, but in

TABLE IX.—*Enzyme Activity of Urine taken from Ureters.*

| No. | Sex. Age. | Date of specimen. | Volume per 24 hour. | Glucuronidase. Units/ml. | Sulphatase. Units/ml. | Ureter. | Operation and date. |
|---|---|---|---|---|---|---|---|
| 1 | F, 57 | 5.v.54 | 160 | 1·37 | 0·57 | Both | Cystectomy 19.iv.54. |
| | | 9.v.54 | 1000 | 0·15 | 0·13 | ,, | |
| 2 | F, 80 | 9.ii.54 | 800 | 1·05 | 1·2 | Right | Ureterostomy and nephrectomy 8.i.54. |
| 3 | M, 76 | 9.iv.54 | 1800 | 1·4 | 0·44 | Pre-operative | Partial cystectomy 11.iv.54. |
| | | 20.iv.54 | 1100 | 0·35 | — | Left | |
| | | | 2000 | 0·36 | — | Right | |
| | | 25.iv.54 | 1600 | 0·43 | 0·87 | Both | |
| 4 | M, 63 | 10.iii.54 | 1200 | 1·2 | 0·37 | Pre-operative | Bilateral transplantation of ureters into colon 11.iii.54. |
| | | 15.iii.54 | 800 | 1·2 | 0·37 | Both | |
| 5 | M, 56 | 11.iii.54 | 4040 | 0·55 | 0·46 | Pre-operative | Right ureteric transplantation. |
| | | 12.iii.54 | 4800 | 0·53 | 0·61 | ,, | |
| | | 15.iii.54 | 1400 | 1·2 | 2·5 | Right | |
| | | 19.iii.54 | 750 | 1·8 | 0·49 | ,, | |
| | | | 1300 | 0·03 | 1·33 | Left through bladder | |
| 6 | M, 57 | 12.vii.54 | 2700 | 1·56 | 0·88 | Pre-operative | Cystectomy 14.vii.54. |
| | | 13.vii.54 | 3500 | 1·32 | 0·66 | Pre-operative | |
| | | 16.vii.54 | 250 | 0·36 | 1·7 | Left | |
| | | | 150 | 0·60 | 2·0 | Right | |
| | | 17.vii.54 | 175 | 0·82 | 2·7 | Left | |
| | | | 80 | 2·0 | 2·4 | Right | |
| | | 18.vii.54 | 510 | 3·1 | 0·57 | Left | |
| | | | 250 | 4·2 | 2·4 | Right | |
| | | 19.vii.54 | 100 | 4·3 | 0·50 | Left | |
| | | | 240 | 4·9 | 0·36 | Right | |
| | | 20.vii.54 | 850 | 5·2 | 2·8 | Left | |
| | | | 350 | 4·9 | 0·86 | Right | |
| | | 21.vii.54 | 1000 | 2·6 | — | Left | |
| | | | 400 | 4·9 | 2·1 | Right | |
| | | 22.vii.54 | 1100 | 5·4 | 1·9 | Left | |
| | | | 200 | 4·3 | 2·8 | Right | |
| | | 23.vii.54 | 1650 | 2·5 | 1·9 | Left | |
| | | | 500 | 4·5 | 2·4 | Right | |
| | | 24.vii.54 | 450 | 4·0 | 1·8 | Left | |
| | | | 500 | 4·5 | 1·3 | Right | |

general sediments show a very much higher activity of both $\beta$-glucuronidase and sulphatase than equal volumes of the corresponding urines. Cancer of the bladder is most frequently found on the lower part of the bladder in both man and animals, and the increased enzyme concentration associated with the sediment may be responsible, at least in part, for this.

*pH Values of Urines.*

The pH values of different specimens of urine given in Tables III, IV, V, VI, VII, VIII and X show wide variations, with no clear difference between the patients with cancer of the bladder and other patients, or normal subjects. The few alkaline urines found (Tables V, VI and VIII) were infected specimens. Most of the specimens had pH values between 5·0 and 6·0, so that the actual glucuronidase activity would be between 20 and 80 per cent of the activity of the optimal pH of 4·5.

TABLE X.—*Urinary Enzyme Activities of Patients with Cancer of Sites other than Bladder.*

| No. | Sex. | Age. | Site of primary tumour. | Volume/24 hours. | Sulphatase. Units/ml. | Glucuronidase. Unit/ml. | Urine pH. |
|---|---|---|---|---|---|---|---|
| 1 | M | 60 | Larynx | 1010 | 0·80 | 0·09 | |
| 2 | F | 43 | Breast | 1650 | 1·30 | 0·09 | |
| 3 | F | 45 | Mylomatosis | 1700 | 0·80 | 0·09 | |
| 4 | F | 68 | Oesophagus | 1890 | 0·40 | 0·09 | |
| 5 | M | 24 | Myeloid leukaemia | 2200 | 0·67 | 0·11 | |
| 6 | F | 54 | Sarcoma thigh | 720 | 0·58 | 0·11 | |
| 7 | M | 66 | Sigmoid colon | 1320 | 0·80 | 0·15 | |
| 8 | F | 54 | Breast | 960 | 0·60 | 0·18 | |
| 9 | M | 74 | Prostate | 1500 | 0·83 | 0·23 | |
| 10 | M | 47 | Colon | 1800 | 1·70 | 0·32 | 5·4 |
| 11 | F | — | Larynx | 650 | 0·90 | 0·33 | |
| 12 | F | 43 | Kidney | 1750 | 0·66 | 0·42 | |
| 13 | F | 76 | Lymphatic leukaemia | 1760 | 0·40 | 0·43 | |
| 14 | M | 37 | Testicle | 700 | 1·39 | 0·46 | 5·3 |
| | | | | 300 | 1·15 | 0·60 | 5·4 |
| 15 | M | 29 | Testicle | 900 | 0·68 | 0·65 | 5·8 |
| | | | | 1400 | 1·08 | 0·80 | 5·4 |
| 16 | M | 75 | Lung | 500 | 2·5 | 0·78 | |
| 17 | M | 66 | Prostate | 1100 | 0·74 | 0·79 | |
| 18 | M | 56 | Stomach | 1080 | 2·4 | 0·84 | |
| 19 | M | 39 | Testicle | 1870 | 3·5 | 0·98 | |
| 20 | M | 57 | Prostate | 2600 | 0·69 | 0·99 | |
| 21 | M | 31 | Testicle | 2100 | 1·73 | 1·35 | 6·7 |
| | | | | 2100 | 1·69 | 1·39 | 7·0 |
| 22 | M | 18 | Testicle | 900 | 2·28 | 1·35 | 5·8 |
| | | | | 1500 | 2·78 | 1·54 | 5·4 |

TABLE XI.—*Urinary Enzyme Activities of Patients with Tuberculosis.*

| No. | Sex. | Age. | Site of disease. | Volume/24 hours. | Sulphatase. Units/ml. | Glucuronidase. Units/ml. |
|---|---|---|---|---|---|---|
| 1 | M | 52 | Lung | 1600 | 0·72 | 0·29 |
| 2 | F | 43 | ,, | 1900 | 1·20 | 0·29 |
| 3 | M | 41 | ,, | 1600 | 0·68 | 0·30 |
| 4 | M | 49 | ,, | 1750 | 1·20 | 0·45 |
| 5 | M | 32 | Kidney | 2500 | 0·35 | 0·60 |
| 6 | M | 58 | ,, | 2520 | 0·80 | 0·63 |
| 7 | F | 37 | Lung | 1250 | 2·90 | 0·68 |
| 8 | M | 72 | ,, | 1000 | 0·92 | 0·75 |
| 9 | M | 36 | Kidney | 1800 | 0·70 | 0·84 |
| 10 | M | 42 | ,, | 1430 | 0·45 | 0·96 |
| 11 | M | 49 | ,, | 1790 | 0·60 | 1·1 |
| 12 | M | 52 | ,, | 1800 | 0·90 | 1·1 |

*Enzyme Activity in Serum.*

The enzyme activity in normal human serum (Table XII) ranged from 0·75–2·5 $\beta$-glucuronidase units per ml. and from 1·2–3·3 sulphatase units per ml. Table XIII gives results for 12 patients with single tumours and for 21 patients with multiple tumours of the bladder. There appears to be an increase in the sulphatase activity compared with the normal values. In all except 5 cases in each of the 2 groups the $\beta$-glucuronidase values are higher than the normal values.

*Enzyme Activity of Malignant and Non-Malignant Bladder Tissues.*

The enzyme activities of the bladder mucosa and bladder tissue tumour taken from patients at operation (Table XIV) show that both types of tissue contained

TABLE XII.—*Enzyme Activity of Serum from Patients free from Malignant Disease.*

| No. | Age. | Sex. | Serum sulphatase. Units/ml. | Serum glucuronidase. Units/ml. |
|---|---|---|---|---|
| 1 | 61 | M | — | 0·75 |
| 2 | 56 | M | — | 0·79 |
| 3 | 76 | M | 1·6 | 1·0 |
| 4 | 52 | M | 1·2 | 1·4 |
| 5 | 58 | F | 2·1 | 1·6 |
| 6 | 62 | M | 3·3 | 1·7 |
| 7 | 85 | F | 1·5 | 1·8 |
| 8 | 65 | M | 1·7 | 1·9 |
| 9 | 55 | M | 1·4 | 2·1 |
| 10 | 62 | M | 2·7 | 2·3 |
| 11 | 50 | M | 1·8 | 2·5 |
| 12 | 36 | M | 1·4 | 2·5 |

TABLE XIII.—*Enzyme Activity of Serum from Patients with Cancer of the Bladder.*

Patients with Single Tumours.

| No. | Age. | Sex. | Serum sulphatase. Units/ml. | Serum glucuronidase. Units/ml. |
|---|---|---|---|---|
| 1 | 39 | M | — | 1·4 |
| 2 | 64 | M | 2·6 | 1·5 |
| 3 | 40 | M | — | 2·0 |
| 4 | 72 | M | — | 2·0 |
| 5 | 61 | M | 2·3 | 2·4 |
| 6 | 65 | M | — | 2·7 |
| 7 | 41 | M | 2·9 | 3·3 |
| 8 | 71 | M | — | 3·8 |
| 9 | 68 | M | 3·1 | 4·1 |
| 10 | 81 | F | 1·9 | 4·8 |
| 11 | 52 | M | 1·1 | 6·0 |
| 12 | 59 | M | 2·2 | 7·5 |

Patients with Multiple Tumours.

| No. | Age. | Sex. | Serum sulphatase. Units/ml. | Serum glucuronidase. Units/ml. |
|---|---|---|---|---|
| 1 | 54 | M | — | 2·0 |
| 2 | 47 | M | — | 2·1 |
| 3 | 68 | M | 3·2 | 2·1 |
| 4 | 66 | M | — | 2·1 |
| 5 | 82 | M | 1·8 | 2·2 |
| 6 | 57 | M | — | 2·7 |
| 7 | 55 | M | 2·4 | 2·8 |
| 8 | 57 | M | 3·6 | 2·9 |
| 9 | 59 | M | 1·5 | 3·0 |
| 10 | 57 | M | — | 3·0 |
| 11 | 54 | M | 2·4 | 3·2 |
| 12 | 47 | M | 1·7 | 3·3 |
| 13 | 57 | M | — | 3·5 |
| 14 | 46 | M | — | 3·6 |
| 15 | 67 | M | 2·0 | 3·7 |
| 16 | 66 | M | — | 4·0 |
| 17 | 54 | M | 1·65 | 4·8 |
| 18 | 42 | M | — | 6·4 |
| 19 | 73 | M | — | 7·9 |
| 20 | 73 | M | 2·9 | 10·7 |
| 21 | 71 | M | — | 10·8 |

TABLE XIV.—*Enzyme Activities of Bladder Tumour and Adjacent Bladder Mucosa.*

| No. | Age. | Sex. | Glucuronidase (units/ml.). Tumour. | Glucuronidase (units/ml.). Mucosa. | Sulphatase (units/ml.). Tumour. | Sulphatase (units/ml.). Mucosa. |
|---|---|---|---|---|---|---|
| 1 | 73 | M | 4·1 | — | 2·1 | — |
| 2 | 59 | M | 9·4 | — | 116·0 | — |
| 3 | 63 | M | 12·5 | 12·0 | 17·0 | 8·2 |
| 4 | 87 | M | 19·0 | 11·0 | 41·0 | 11·0 |
| 5 | 39 | M | 25·0 | 9·0 | 23·0 | 2·7 |
| 6 | 71 | M | 26·0 | 55·7 | 14·6 | 15·0 |
| 7 | 61 | M | 29·0 | 61·0 | 54·0 | 10·0 |
| 8 | 59 | M | 34·0 | 29·0 | 48·0 | 5·2 |
| 9 | 50 | M | 43·0 | 35·0 | 41·0 | 11·0 |
| 10 | 72 | M | 44·0 | 55·0 | 48·0 | 9·0 |
| 11 | 72 | M | 47·0 | 25·0 | 107·0 | 10·0 |
| 12 | 54 | M | 50·0 | — | 25·0 | — |
| 13 | 69 | F | 55·0 | — | 41·0 | — |
| 14 | 63 | M | 55·0 | 12·0 | 44·0 | 11·0 |
| 15 | 42 | M | 58·0 | — | 400·0 | — |
| 16 | 72 | M | 58·0 | — | 41·0 | — |
| 17 | 71 | M | 72·0 | 55·0 | 32·0 | 15·0 |
| 18 | 45 | M | 77·0 | — | 50·0 | — |
| 19 | 64 | F | 79·0 | — | 128·0 | — |
| 20 | 59 | M | 91·0 | — | 40·0 | — |
| 21 | 69 | M | 96·0 | 73·0 | 83·0 | 12·0 |
| 22 | 45 | M | 97·0 | — | 7·4 | — |
| 23 | 67 | F | 106·0 | 61·0 | — | 0·6 |
| 24 | 57 | M | 112·0 | 40·0 | 46·0 | 16·0 |
| 25 | 61 | M | 120·0 | 7·5 | 31·0 | 32·0 |
| 26 | 56 | F | 140·0 | 105·0 | 78·0 | 16·0 |
| 27 | 50 | M | 145·0 | 57·0 | 26·0 | 26·0 |
| 28 | 67 | M | 156·0 | — | 82·0 | — |
| 29 | 74 | M | 170·0 | — | 116·0 | — |
| 30 | 58 | M | 176·0 | — | 61·0 | — |
| 31 | 64 | F | 255·0 | 235·0 | 240·0 | 11·0 |
| 32 | 71 | M | 455·0 | — | 390·0 | — |

β-glucuronidase and sulphatase. In most of the specimens, the tumour tissue had more enzyme activity than the corresponding normal mucosa. Fishman and Anylan (1947) found that other types of human cancer contained more β-glucuronidase than the adjacent normal tissue.

### DISCUSSION.

The enzyme excretions have been expressed as activities per unit volume of urine, but the volume of the urine was always recorded, and the results have also been calculated as total units of enzyme excreted per day. When expressed in this way, the results were essentially the same except that the bladder cancer patients gave higher values because such patients are usually encouraged to take large volumes of fluid and so excrete more urine than other subjects. The method of expressing the results which is used weights the result against the difference which has been found, namely increased enzyme excretion in cancer patients.

The investigations would be facilitated if a means of measuring the increased excretion could be used which would avoid the collection of the 24-hour specimen. The ratio of enzyme activity to some urinary constituent or to the total urinary constituents, as indicated by the specific gravity, might serve this purpose. This problem and the investigation of $\beta$-glucuronidase in sera and urines of patients with cancer of sites other than the bladder are being investigated.

The data presented show that patients with cancer of the bladder have higher urinary $\beta$-glucuronidase and sulphatase concentrations than those subjects free of malignant disease. Most of the bladder cancer patients examined excrete large volumes of urine so that the total enzyme excretion in such cases is generally larger than in other subjects. This finding is in agreement with the hypothesis that the urinary $\beta$-glucuronidase is a contributing cause of the disease, acting by liberation of carcinogenic aminophenols or other compounds from inactive conjugates. On the other hand, the raised urinary $\beta$-glucuronidase may be a result of the disease rather than the cause. In the few cases in which specimens of urine were taken directly from the ureter, the $\beta$-glucuronidase and sulphatase activities were of the same order as it is in bladder urine; the extra $\beta$-glucuronidase is unlikely to have its origin in the bladder although normal bladder mucosa usually had a higher concentration of $\beta$-glucuronidase than urine. The urinary $\beta$-glucuronidase was generally raised even in patients with no active tumour. The bladder tumours like many other malignant tumours, usually had a higher sulphatase and $\beta$-glucuronidase content than the normal tissue from which the tumour was derived.

Although the serum enzyme values of cancer patients were generally above the normal range, the distinction between the cancer and cancer-free patients was much less clear cut than was the case with the urinary enzymes. The urinary enzymes are probably derived from the blood; the amount of the enzymes in urine being dependent on the serum concentration and the degree to which protein, including enzymes, pass through the kidney. The enzymes may pass from the kidney itself directly into urine, as kidney tissue contains both $\beta$-glucuronidase and sulphatase, but the fact that the enzyme activity of urine from the two kidneys at any one time was the same would indicate that the enzymes were derived from circulating blood rather than kidney tissue.

Other work on the action of enzymes on derivatives of the carcinogenic 2-amino-1-hydroxynaphthalene (Boyland, Manson, Sims and Williams, 1955) has shown that whereas 2-amino-1-hydroxynaphthalene phosphoric ester and glucuronide are hydrolysed by phosphatase and $\beta$-glucuronidase respectively, 2-amino-1-hydroxynaphthalene sulphuric ester is not hydrolysed by the sulphatases of human urine, rat kidney or takadiastase. This would indicate that sulphatase could not play any rôle in carcinogenesis from $\beta$-naphthylamine, as the 2-amino-1-hydroxynaphthalene sulphuric ester would not be hydrolysed in urine. $\beta$-Glucuronidase can, however, release the carcinogenic 2-amino-1-hydroxynaphthalene from its glucuronide which Boyland and Manson (unpublished observations) have shown to be a metabolite of $\beta$-naphthylamine.

Although the level of the urinary enzymes may be of value in prognosis, in as much as it may indicate the liability of fresh tumours to develop, the patients who have been investigated so far over the last year, with different types of tumour and different types of treatment, have not been followed up long enough to make any definite statement as to the value of the estimations in prognosis.

### SUMMARY.

(1) Urinary sulphatase activity of patients with cancer of the bladder is often high compared with normals. This rise may be due in part to infection. Urine sediments from bladder cancer patients are always high in sulphatase activity but serum sulphatase is not raised in cancer patients.

(2) $\beta$-glucuronidase activity is almost always increased in the urine of patients with cancer of the bladder and remains high in most cases even after the tumour has been removed. The $\beta$-glucuronidase activity of urine is independent of infection of the urine. A few patients suffering from other forms of cancer had slightly increased urinary $\beta$-glucuronidase.

(3) The $\beta$-glucuronidase activity is high in all urinary sediment examined and in most sera from patients suffering from cancer of the bladder.

(4) Bladder-cancer tissue contained more $\beta$-glucuronidase and sulphatase than the corresponding bladder mucosa.

(5) The estimation of $\beta$-glucuronidase activity in urine may be useful for prognostic purposes in bladder cancer.

We are indebted to Dr. J. M. O. Earle for the cell counts of urine specimens and to Mr. P. L. Grover for technical assistance.

This investigation has been supported by grants to the Chester Beatty Research Institute, Institute of Cancer Research : Royal Cancer Hospital from the British Empire Cancer Campaign, the Jane Coffin Childs Memorial Fund for Medical Research, the Anna Fuller Fund, and the National Cancer Institute of the National Institutes of Health, U.S. Public Health Service.

### REFERENCES.

Bonser, G. M.—(1943) *J. Path. Bact.*, **55**, 1.
*Idem.*, Clayson, D. B., Jull, J. W. and Pyrah, L. M.—(1952) *Brit. J. Cancer*, **6**, 412.
Boyland, E., Manson, D., Sims, P. and Williams, D. C.—(1955) *Biochem. J.* (In press.)
Dzialoszynski, L. M.—(1950) *Nature*, **160**, 464.
Fishman, W. H. and Anlyan, A. J.—(1947) *Cancer Res.*, **7**, 808.
*Idem.*, Springer, B. and Brunetti, J.—(1948) *J. biol. Chem.*, **173**, 449.
Hueper W. C., Wiley, F. H. and Wolfe, H. D.—(1938) *J. industr. Hyg.*, **20**, 46.
*Idem.*, and Wolfe, H. D.—(1937) *Amer. J. Path.*, **13**, 656.
Huggins, C. and Smith, D. R.—(1947) *J. biol. Chem.*, **170**, 391.
Robinson, D., Smith, J. N., Spencer, B. and Williams, R. T.—(1952) *Biochem. J.*, **51**, 202.
*Idem.*, Smith, J. N. and Williams, R. T.—(1951) *Ibid.*, **49**, IXXIV.
Roy, A. B.—(1953) *Ibid.*, **53**, 12.
Talalay, F., Fishman, W. H. and Huggins, C.—(1946) *J. biol. Chem.*, **166**, 757.
Tanaka, S.—(1938) *J. Biochem., Tokyo*, **28**, 119.